# Diverse alternative back-splicing and alternative splicing landscape of circular RNAs

**DOI:** 10.1101/gr.202895.115

**Published:** 2016-09

**Authors:** Xiao-Ou Zhang, Rui Dong, Yang Zhang, Jia-Lin Zhang, Zheng Luo, Jun Zhang, Ling-Ling Chen, Li Yang

**Affiliations:** 1 Key Laboratory of Computational Biology, CAS Center for Excellence in Brain Science and Intelligence Technology, CAS-MPG Partner Institute for Computational Biology, Shanghai Institutes for Biological Sciences, Chinese Academy of Sciences, Shanghai 200031, China;; 2 University of Chinese Academy of Sciences, Beijing 100049, China;; 3 State Key Laboratory of Molecular Biology, CAS Center for Excellence in Molecular Cell Science, Institute of Biochemistry and Cell Biology, Shanghai Institutes for Biological Sciences, Chinese Academy of Sciences, Shanghai 200031, China;; 4 School of Life Science, ShanghaiTech University, Shanghai 20003, China

## Abstract

Circular RNAs (circRNAs) derived from back-spliced exons have been widely identified as being co-expressed with their linear counterparts. A single gene locus can produce multiple circRNAs through alternative back-splice site selection and/or alternative splice site selection; however, a detailed map of alternative back-splicing/splicing in circRNAs is lacking. Here, with the upgraded CIRCexplorer2 pipeline, we systematically annotated different types of alternative back-splicing and alternative splicing events in circRNAs from various cell lines. Compared with their linear cognate RNAs, circRNAs exhibited distinct patterns of alternative back-splicing and alternative splicing. Alternative back-splice site selection was correlated with the competition of putative RNA pairs across introns that bracket alternative back-splice sites. In addition, all four basic types of alternative splicing that have been identified in the (linear) mRNA process were found within circRNAs, and many exons were predominantly spliced in circRNAs. Unexpectedly, thousands of previously unannotated exons were detected in circRNAs from the examined cell lines. Although these novel exons had similar splice site strength, they were much less conserved than known exons in sequences. Finally, both alternative back-splicing and circRNA-predominant alternative splicing were highly diverse among the examined cell lines. All of the identified alternative back-splicing and alternative splicing in circRNAs are available in the CIRCpedia database (http://www.picb.ac.cn/rnomics/circpedia). Collectively, the annotation of alternative back-splicing and alternative splicing in circRNAs provides a valuable resource for depicting the complexity of circRNA biogenesis and for studying the potential functions of circRNAs in different cells.

Circular RNAs (circRNAs) formed by exon back-splicing (circularization) were originally identified in the 1990s (Nigro et al. 1991; Capel et al. 1993). Recently, circRNAs were rediscovered and shown to be the products of thousands of loci in eukaryotes, from fly and worm to mouse and human (Jeck et al. 2013; Memczak et al. 2013; Salzman et al. 2013; Guo et al. 2014; Westholm et al. 2014; Zhang et al. 2014; Ivanov et al. 2015). Recent research into circRNA biogenesis has shown that back-splicing is catalyzed, though inefficiently (Zhang et al. 2016), by the canonical spliceosomal machinery (Ashwal-Fluss et al. 2014; Starke et al. 2015; Wang and Wang 2015) and modulated by both *cis*-elements and *trans*-factors (Ashwal-Fluss et al. 2014; Zhang et al. 2014; Conn et al. 2015; Ivanov et al. 2015; Starke et al. 2015; for review, see Chen and Yang 2015; Chen 2016). Different from the canonical splicing that joins an upstream 5′ splice (donor) site with a downstream 3′ splice (acceptor) site in a sequential order to produce a linear RNA, back-splicing occurs in a reversed orientation that links a downstream 5′ splice (donor) site to an upstream 3′ splice (acceptor) site to yield a circRNA. Thus, the identification of back-splice junctions is crucial to annotate circRNAs (Jeck and Sharpless 2014).

With the intrinsic feature of being covalently closed without open ends, circRNAs are largely missed by polyadenylated transcriptome profiling but can be captured by RNA deep-sequencing (RNA-seq) from nonpolyadenylated RNAs (Yang et al. 2011; Jeck et al. 2013; Memczak et al. 2013; Salzman et al. 2013; Zhang et al. 2014). Two nonpolyadenylated RNA isolation strategies have been applied to RNA-seq to retrieve back-splice junction reads for circRNA annotation. On the one hand, nonpolyadenylated RNAs can be co-collected with polyadenylated transcripts after depleting ribosomal RNAs (ribo^−^) (Memczak et al. 2013). On the other hand, the relatively purer nonpolyadenylated RNA fractionation can be enriched by depleting both ribosomal RNAs and polyadenylated RNAs (poly(A)^−^/ribo^−^, or p(A)^−^ for simplicity) (Yang et al. 2011; Zhang et al. 2014). By counting back-splice junction reads, the expression of individual circRNA can be quantitatively evaluated (Memczak et al. 2013; Zhang et al. 2014).

Interestingly, a single gene locus can produce multiple circRNAs through alternative back-splicing (circularization) by a mechanism associated with the competition of putative RNA pairs across introns that bracket the circle-forming exons (Zhang et al. 2014). Theoretically, there are two types of alternative back-splicing (Fig. 1A). One type is alternative 5′ back-splicing, in which two or more 5′ downstream back-splice sites alternatively link to the same upstream 3′ back-splice site in a reversed orientation. The other one is alternative 3′ back-splicing, in which two or more upstream 3′ back-splice sites alternatively link to the same downstream 5′ back-splice site in a reversed orientation. Apparently, alternative back-splicing further expands the complexity of circRNA formation; however, the detailed annotation of alternative back-splicing is largely unknown.

**Figure 1.**
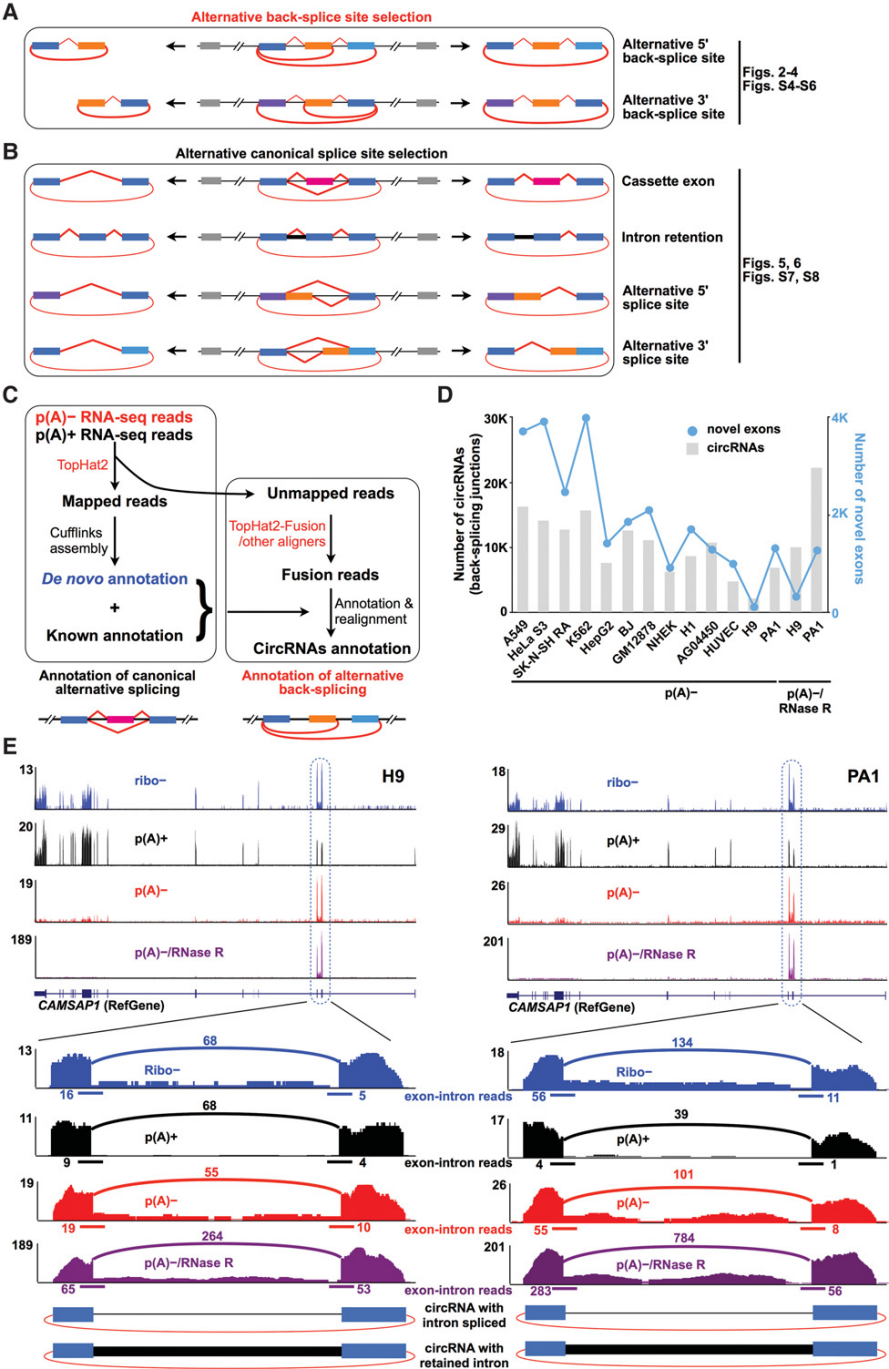
An upgraded computational pipeline (CIRCexplorer2) to systematically identify alternative (back-)splicing in back-spliced circular RNAs (circRNAs). (*A*) Schematic diagrams of two types of alternative back-splicing. Colored bars, exons. Black lines, introns. Red polylines, (canonical) collinear splicing. Red arc lines, back-splicing (circularization). (*B*) Schematic diagrams of four basic types of alternative splicing. Colored bars, exons. Black lines, introns. Red lines, splicing. Red arc lines, back-splicing (circularization). (*C*) The schematic diagram of CIRCexplorer2. The analysis was performed as described (Zhang et al. 2014) with modifications (Supplemental Methods). Alternative back-splicing and alternative splicing in circRNAs were determined with stringent criteria (Supplemental Methods). (*D*) Ten thousand circRNAs (gray bars) were detected by CIRCexplorer2. Thousands of novel exons (blue points) were identified in circRNAs in different human cell lines with de novo assembly (Supplemental Methods). (*E*) The identification and visualization of circRNAs in the *CAMSAP1* locus from H9 (*left* panel) or PA1 (*right* panel) cell lines. Different types of RNA-seq data sets from ribo^−^, p(A)^+^, p(A)^−^ or p(A)^−^/RNase R RNA populations were used for comparison. *CAMSAP1* circRNAs could be determined from ribo^−^, p(A)^−^, and p(A)^−^/RNase R RNA-seq data sets by identifying back-splice junctions. Notably, ribo^−^ RNA-seq is not suitable to study canonical splicing events (intron retention, in this case) that occur specifically within circRNAs, as ribo^−^ RNAs contain both polyadenylated and nonpolyadenylated transcripts. Blues bars, exons. Black lines, introns. Black thick line, the retained intron. Red arc lines, back-splicing (circularization).

Because the majority of annotated human circRNAs consist of multiple exons (Zhang et al. 2014), alternative splicing, in principle, should be yet another mechanism that can enlarge the diversity of circRNAs. It is well known that most multiexonic genes undergo alternative splicing to generate multiple (linear) mRNAs (Nilsen and Graveley 2010). Thus, alternative splicing can also expand the diversity of circRNAs. For example, some cassette exons are more favorably included in circRNAs than in linear mRNAs (Zhang et al. 2014), and some introns are retained in circRNAs (Salzman et al. 2013; Zhang et al. 2014; Li et al. 2015) through alternative splicing. Nevertheless, the precise annotation of alternative splicing within circRNAs is unclear, and the degree to which the difference in the alternative splicing pattern between circRNAs and their correlated linear counterparts remains to be investigated.

We hereby applied CIRCexplorer2, an upgraded computational pipeline, to identify both alternative back-splicing (Fig. 1A) and alternative splicing (Fig. 1B) events in circRNAs from various p(A)^−^ RNA-seq data sets. Through a comparison with parallel poly(A)^+^ (p(A)^+^ for simplicity) RNA-seq data sets in the same cell lines, thousands of alternatively back-spliced and circRNA-predominant alternatively spliced exons, including previously unannotated ones, were identified in circRNAs with variable expression patterns from different p(A)^−^ and p(A)^−^/RNase R RNA-seq data sets. Together, the diverse landscape of alternative back-splicing and alternative splicing in circRNAs provides a valuable resource for depicting the complexity of circRNA formation.

## Results

### An upgraded computational pipeline for circRNA annotation

Multiple computational methods have been recently developed to detect back-splice junctions for circRNA annotation (Memczak et al. 2013; Hoffmann et al. 2014; Westholm et al. 2014; Zhang et al. 2014). Our previously reported pipeline, CIRCexplorer (Zhang et al. 2014), has been reported as one of the best pipelines for circRNA annotation by identifying back-splice junctions (Hansen et al. 2016). To systematically identify both alternative back-splice junctions (Fig. 1A) and alternative splice junctions (Fig. 1B) in circRNAs, we upgraded our pipeline to CIRCexplorer2 (Fig. 1C; Methods). Several major improvements have been implemented in the upgraded pipeline. First, according to requests from many users, we have incorporated additional aligners, such as STAR (Dobin et al. 2013), MapSplice (Jeck et al. 2013), and segemehl (Hoffmann et al. 2014), to fit the different requirements/preferences of RNA-seq mapping. All of these aligners in CIRCexplorer2 can be used to identify back-splicing with similar outcomes (Supplemental Fig. S1A,B). It is worth noting, however, that combining different aligners might provide a better prediction of back-splicing/circularization (Zhang et al. 2014; Hansen et al. 2016). Second, the p(A)^−^ RNA-seq reads that mapped to the genome and the collinear exon-exon junctions were not simply discarded but were instead further de novo assembled for the identification of novel exons and novel splicing events. Finally, TopHat-unmapped but TopHat-Fusion-mapped reads were realigned to both known and de novo assembled annotations to determine back-splice junctions from either annotated and/or novel exons (Fig. 1C; Supplemental Methods). The false discovery rate of the upgraded CIRCexplorer2 pipeline remains at the low level (Supplemental Fig. S1C) when checked with the reported strategy (Hansen et al. 2016).

### Detection of alternative back-splicing/splicing in circRNAs from p(A)^−^ RNA-seq data sets

Next, we applied CIRCexplorer2 to identify both alternative back-splicing and alternative splicing in circRNAs from various p(A)^−^ RNA-seq data sets of human cell lines, including the human embryonic stem cell (hESC) H9 line, human ovarian carcinoma PA1 cells, and 11 ENCODE cell lines (Methods). These data sets contain tens of thousands to hundreds of millions of RNA-seq reads (Supplemental Table S1). Parallel p(A)^+^ RNA-seq data sets were used to discriminate circRNA-specific/-predominant alternative splicing from the linear RNA counterparts. The p(A)^−^/RNase R RNA-seq data sets from both H9 and PA1 cells were generated in the laboratory to confirm that the detected alternative back-splicing and alternative splicing events were from circRNAs but not from their linear counterparts.

In total, more than 10,000 alternative back-splicing and alternative splicing events were identified in at least one of the examined cell lines (Fig. 1D; Supplemental Table S2). Strikingly, with an additional de novo assembly step in the updated CIRCexplorer2, thousands of novel exons were detected in circRNAs from examined cell lines (Fig. 1D). The identified alternative back-splicing and alternative splicing can be visualized at CIRCpedia (http://www.picb.ac.cn/rnomics/circpedia) (Supplemental Fig. S2).

Although ribo^−^ RNA-seq data sets were used for circRNA annotation, such data sets were not suitable for the analysis of circRNA-alternative splicing. Because both polyadenylated and nonpolyadenylated transcripts are included in the ribo^−^ RNA population, it is impractical to discriminate whether the identified alternative splicing events are from linear (m)RNAs or circRNAs (Fig. 1E; Supplemental Fig. S3). Thus, in the current study, only p(A)^−^ and p(A)^−^/RNase R treated RNA-seq data sets were used to profile alternative back-splicing and alternative splicing in circRNAs.

### Landscape of alternative back-splicing in circRNAs

**Figure 2.**
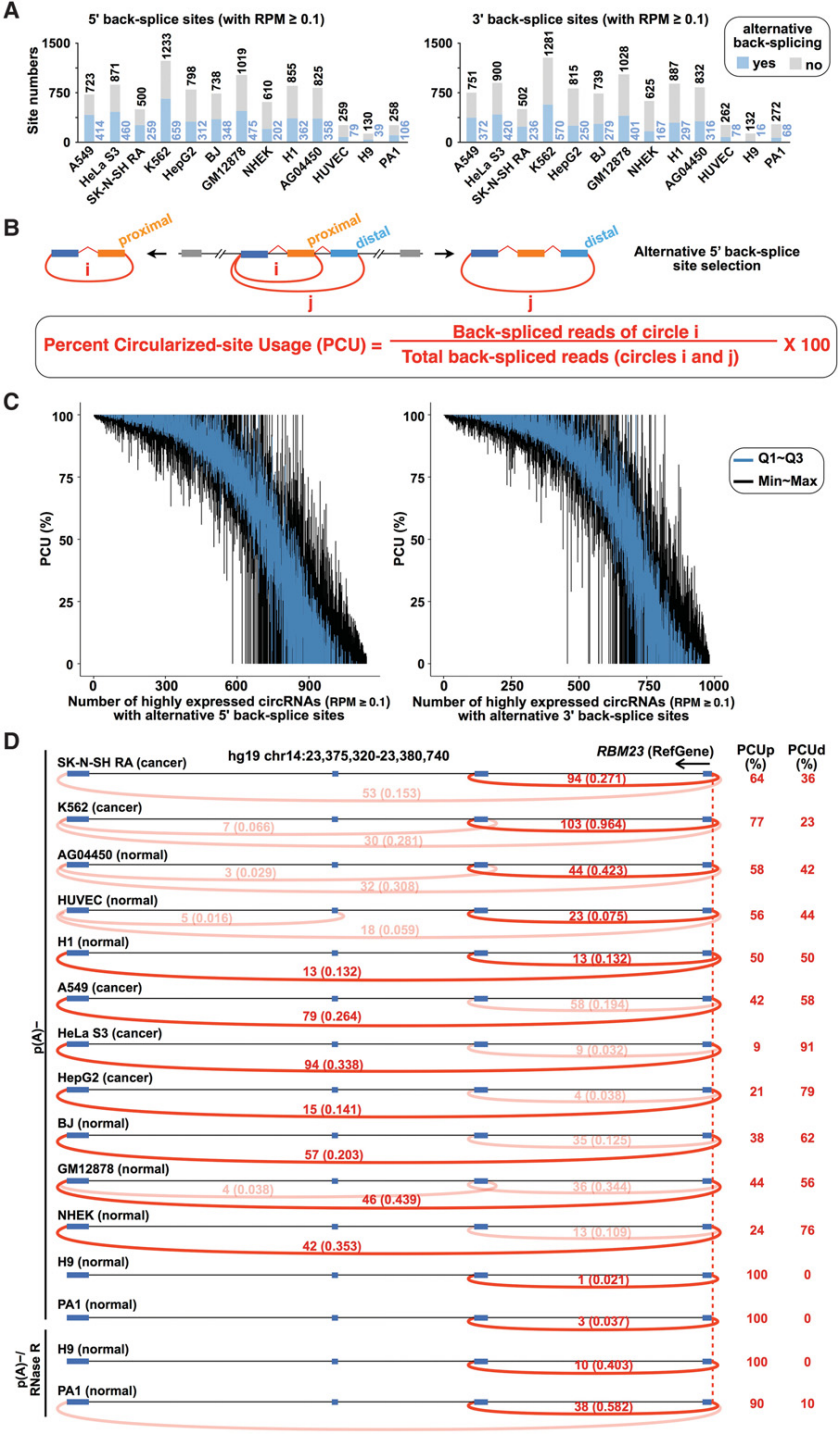
The diverse landscape of alternative back-splicing. (*A*) Approximately 12%–57% of back-splice sites are alternatively selected among high-confidence expressed circRNAs with RPM (mapped back-splice junction Reads Per Million mapped reads) ≥ 0.1. (*B*) A schematic diagram of alternative 5′ back-splicing and its quantification (*top* panel). The use of proximal and distal 5′ back-splice sites can be quantitated by the Percent Circularized-site Usage (PCU, *bottom* panel) with detected back-splice junction reads (i and j, respectively). (*C*) Diverse usage of alternative 5′ (*left*) and 3′ (*right*) back-splice sites among different cell lines. Each blue vertical line denotes PCU variation for one circRNA from the first quartile (Q1) to the third quartile (Q3) across cell lines, and each black vertical line denotes PCU variation from the minimum to the maximum. Note that only highly expressed circRNAs with RPM ≥ 0.1 in at least three cell lines were used for this analysis. (*D*) Visualization of alternative 5′ back-splice site usage in circRNAs produced from the *RBM23* locus across different cell lines. Predicted circRNAs in the *RBM23* locus were indicated by red arc lines with raw back-splice junction reads and normalized RPMs (numbers *above* each arc line, *left* panel). The use of proximal alternative 5′ back-splice site (PCUd) or distal alternative 5′ back-splice site (PCUp) was calculated accordingly (*right* panel).

With CIRCexplorer2, more than 10,000 circRNAs were identified, and their downstream 5′ back-splice sites and upstream 3′ back-splice sites were accordingly annotated (Supplemental Fig. S4A). Of the highly expressed circRNAs with mapped back-splice junction reads ≥ 0.1 RPM (mapped back-splice junction Reads Per Million mapped reads) (Zhang et al. 2014), up to 30% were alternatively back-spliced (Fig. 2A; Supplemental Fig. S4B; Supplemental Table S2), which suggests that alternative back-splicing is widely distributed in circRNAs. We further used PCU (Percent Circularized-site Usage) (Methods), the usage of each alternative back-splice site, to evaluate and compare the diverse alternative back-splicing events across samples (Fig. 2B).

In total, up to 70% of the alternative 5′/3′ back-splicing in highly expressed circRNAs was detected in multiple p(A)^−^ RNA-seq data sets (Supplemental Fig. S4C), and the use of these alternative back-splice sites is significantly diverse among the examined cell lines (Fig. 2C). For instance, there are two choices for the alternative 5′ back-splice site selection, i.e., the proximal selection and the distal selection, in the human *RBM23* circRNAs (Fig. 2D). Both events were detected in 12 of the 13 examined cell lines with available p(A)^−^ and/or p(A)^−^/RNase R RNA-seq data sets. However, the use of these two alternative 5′ back-splice sites was largely different among cell lines. The PCU of the proximal alternative 5′ back-splice site (PCUp) varied from 9% to 100%, and, accordingly, the PCU of the distal alternative 5′ back-splice site (PCUd) varied from 91% to almost 0 (right panel, Fig. 2D). Notably, the diverse usages of alternative back-splice sites in highly expressed circRNAs (with RPM ≥ 0.1 in at least three cell lines) were less affected by sequence depths than by their variable expression in different cell lines (Supplemental Fig. S4D).

**Figure 3.**
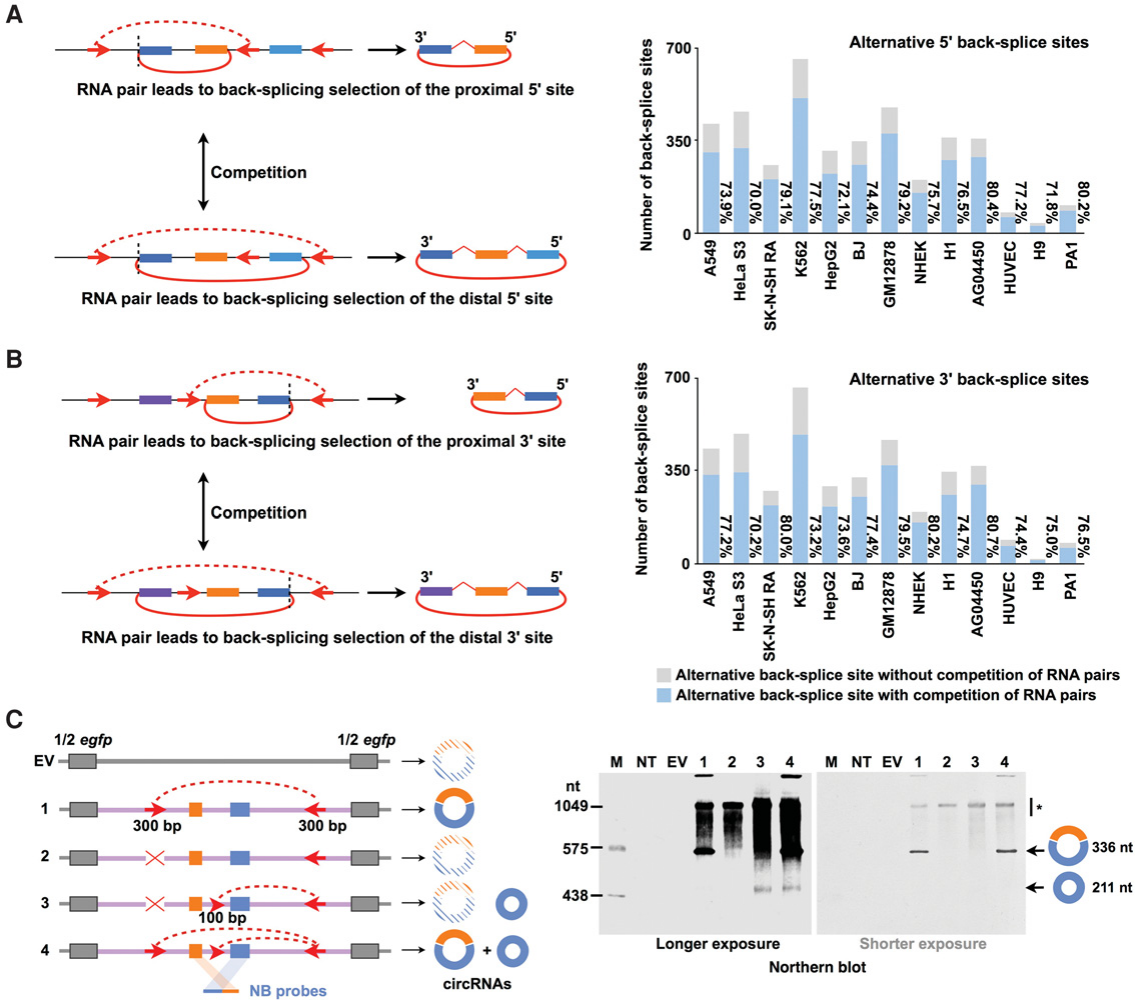
Competition of RNA pairs flanking proximal or distal back-splice sites leads to alternative back-splice site selection. (*A*,*B*) Potential RNA pairs (red dashed arc lines) produced by orientation-opposite complementary sequences (red arrows) flanking proximal (*left top* panels) or distal (*left bottom* panels) back-splice sites lead to alternative 5′ (*A*)/3′ (*B*) back-splice site selection, respectively (red arc lines). The competition of RNA pairs flanking proximal or distal back-splice sites leads to alternative back-splice site selection. More than 70% of the highly expressed circRNAs (RPM ≥ 0.1) with alternative back-splice site selection contain potential paired complementary sequences flanking both proximal and distal 5′/3′ back-splice sites (*right* panels). (*C*) Recapitulation of alternative back-splicing. (*Left*) A schematic drawing of *egfp* expression vectors with engineered complementary sequences for *POLR2A* circular RNA recapitulation. Half *egfp* sequences from the expression vector backbone are indicated as gray bars. *POLR2A* exonic and intronic sequences are indicated as colored bars and light purple lines, respectively. Nonrepetitive complementary sequences (red arrows) were inserted into multiple *POLR2A* intronic regions to form different RNA pairs (red dashed arc lines). Northern blot (NB) probes are indicated as colored bars. (*Right*) Validation of alternatively back-spliced *POLR2A* circRNAs by Northern blot on denaturing PAGE gel. Note that only partial complementary sequence (∼100 bp) was inserted into the middle intron for smaller *POLR2A* circRNA. (*) Linear RNA background.

Since the complementary sequences in flanking introns can promote exon circularization (Liang and Wilusz 2014; Zhang et al. 2014), we evaluated whether the existence of multiple pairs of intronic complementary sequences would have an effect on alternative back-splice site selection. Theoretically, an across-intron RNA pair flanking the proximal back-splice sites would lead to proximal back-splice site selection (left panels of Fig. 3A,B, top). Similarly, an across-intron RNA pair flanking the distal back-splice sites could lead to distal back-splice site selection (left panels of Fig. 3A,B, bottom). Thus, the formation of a proximal RNA pair competes with a distal RNA pair, resulting in the alternative back-splice site selection in a single gene locus. A computational analysis of the orientation-opposite complementary sequences revealed that this scenario is indeed the case. More than 70% of the highly expressed circRNAs (junction reads ≥ 0.1 RPM) with alternative back-splice site selection contained the paired intronic complementary sequences flanking both the proximal and distal 5′/3′ back-splice sites (right panels of Fig. 3A,B); in comparison, ∼20% of randomly selected nonalternative back-splicing events were flanking with paired intronic complementary sequences (Supplemental Fig. S5A,B). Clusters of proximal and distal RNA pairs across introns were seen in many gene loci (for example, the human *RBM23* locus) (Supplemental Fig. S5C,D), and this strong competition between these potential RNA pairs was correlated with the detected alternative back-splicing events (Fig. 2D). This analysis provides yet another line of evidence demonstrating that *cis*-elements can significantly affect the biogenesis of circRNAs.

We next used expression vectors to validate that the competition of paired intronic complementary sequences leads to alternative back-splice site selection. As previously reported (Zhang et al. 2014), paired complementary sequences engineered into the flanking introns could significantly increase the expression of *POLR2A* circular RNA that contains two exons (Fig. 3C, #1). When the paired structure was disrupted, the *POLR2A* circular RNA expression was dramatically reduced to undetectable levels (Fig. 3C, #2). When paired complementary sequences were engineered into the introns flanking only one exon, a smaller *POLR2A* circular RNA with that exon was induced (Fig. 3C, #3). Interestingly, when multiple complementary sequences were individually inserted into different introns that led to the competition of two RNA pairs, both the original *POLR2A* circular RNA with two exons and the smaller *POLR2A* circular RNA with only one exon could be expressed from the same expression vector (Fig. 3C, #4). Alternative back-splice sites of both recapitulated *POLR2A* circRNAs were further confirmed by Sanger sequencing after RT-PCR with divergent primers. However, it should be noted that the competition between intronic RNA pairs and their induced alternative back-splicing regulation could be more complicated under endogenous conditions (Supplemental Fig. S5C).

### Back-splicing with novel exons

**Figure 4.**
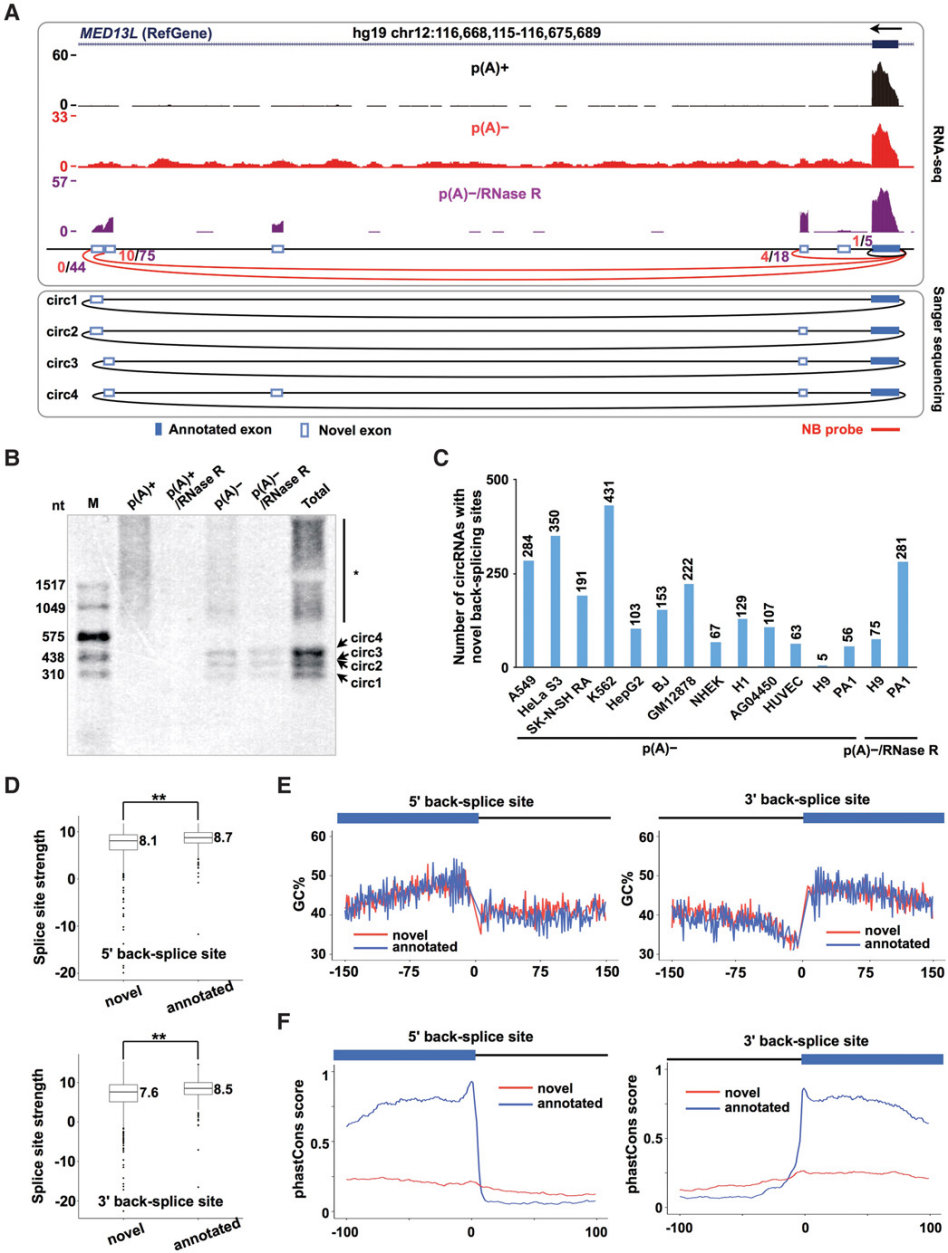
Unannotated exons produced from alternative back-splicing. (*A*) At least four novel exons (white bars) in the human *MED13L* locus were identified in PA1 p(A)^−^ and/or p(A)^−^/RNase R RNA-seq data sets. The predicted circRNAs in the *MED13L* locus were indicated by red arc lines with raw back-splice junction reads from p(A)^−^ (red) and/or p(A)^−^/RNase R (purple) RNA-seq data sets. Alternative back-splice sites were determined by both RNA-seq (*top* panel) and Sanger sequencing (*bottom* panel). Note that these new exons were barely detected in linear counterparts from parallel p(A)^+^ RNA-seq (the wiggle track in black). (*B*) Validation of multiple *MED13L* circRNAs with previously unannotated exons by Northern blot on native agarose gel. Note that the validation of these *MED13L* circRNAs with novel exons was consistent with RT-PCR (Supplemental Fig. S6B). (*) Linear RNA background. (*C*) Hundreds to thousands of circRNAs were identified with novel back-splice sites across different cell lines. (*D*) Splicing strength of novel back-splice sites is comparable to that of annotated back-splice sites. (**) *P* value < 0.01, Wilcoxon rank-sum test. (*E*) Novel (red) and annotated back-spliced exons (blue) have similar GC contents. (*F*) Novel back-spliced exons (red) are less conserved in sequences than are annotated back-spliced exons (blue).

The de novo assembly of the unmapped reads from the p(A)^−^ and/or p(A)^−^/RNase R RNA-seq data sets by the upgraded CIRCexplorer2 pipeline revealed that many alternative 5′/3′ back-splice sites from previously unannotated exons (non-RefSeq, non-UCSC Known Genes, or non-Ensembl) were predominantly detected in circRNAs (Supplemental Table S3). For instance, in the human *MED13L* locus, at least four previously unannotated exons were identified in p(A)^−^ and/or p(A)^−^/RNase R RNA-seq data sets in PA1 and/or other cell lines (Fig. 4A; Supplemental Fig. S6A), and three of the four were alternatively back-spliced, as shown by the identified back-splice junction reads (red arc lines in Fig. 4A). The existence of these novel alternative 5′ back-spliced sites was further confirmed by Sanger sequencing (Fig. 4A, bottom panel) after RT-PCR amplification (Supplemental Fig. S6B) and by Northern blot analysis (Fig. 4B). In contrast, the inclusion of these novel exons was rarely detected in the linear *MED13L* mRNA (Fig. 4A, p(A)^+^ RNA-seq; Supplemental Fig. S6B, RT-PCR). Although with a similar sequence feature (Supplemental Fig. S6C), hundreds of 5′/3′ back-splice sites from previously unannotated exons were identified in circRNAs among different cell lines (Fig. 4C; Supplemental Table S3) with at least one back-splice junction read from available p(A)^−^ RNA-seq data sets but were largely missed in linear RNAs (Supplemental Fig. S6D).

These novel back-splice sites have a slightly lower splicing strength than the randomly selected annotated exons (Fig. 4D). In addition, such novel back-splice sites in general contain similar sequence signatures to those of annotated exons (Fig. 4E). Interestingly, our analysis further revealed that these novel exons are less conserved in sequence than annotated exons (Fig. 4F). As it has been suggested that back-splicing is unfavorably processed by the spliceosome (Jeck and Sharpless 2014; Chen and Yang 2015; Starke et al. 2015; Zhang et al. 2016), it is unclear how the spliceosome could specifically recognize these exons during back-splicing but not during canonical splicing. In this case, novel mechanisms that are associated with back-splicing await discovery.

### The complexity of alternative splicing within circRNAs

The majority of the annotated human circRNAs consist of multiple exons (Zhang et al. 2014), indicating that potential alternative splicing events could occur during circRNA formation. With the upgraded CIRCexplorer2 pipeline and available p(A)^−^ and p(A)^−^/RNase R RNA-seq data sets, all four basic types of alternative splicing were identified within the circRNAs from the examined cell lines (Supplemental Fig. S7). We further quantitated the extent of different types of alternative splicing by PSI (Percent Spliced In) for cassette exon selection, PIR (Percent Intron Retention) for intron retention, and PSU (Percent Splice-site Usage) for alternative 5′/3′ splice site selection. All of the splicing events with more selection in circRNAs than those in their linear cognates were counted (Supplemental Table S4; Supplemental Methods). These analyses revealed that 20%–30% of the circRNA-specific/-predominant alternative splicing events could be detected in multiple examined cell lines (Supplemental Fig. S7).

**Figure 5.**
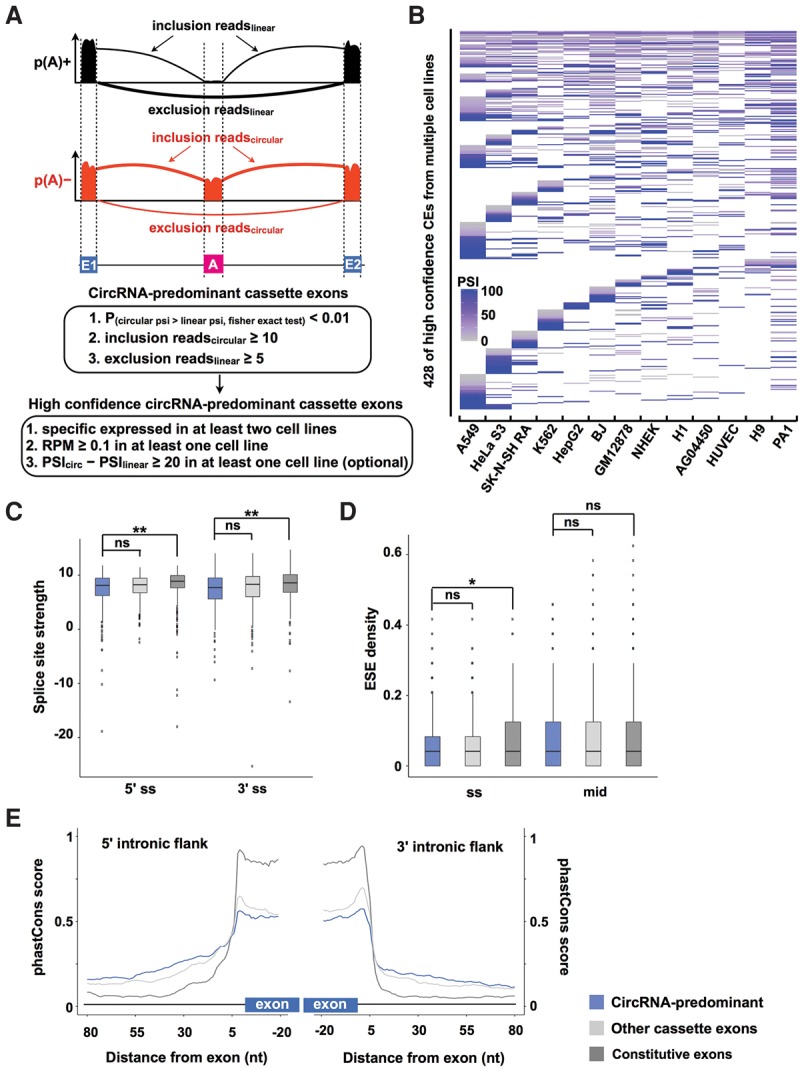
Characterization of circRNA-predominant alternative cassette exons. (*A*) A strategic pipeline to identify high-confidence circRNA-predominant alternative cassette exons. By comparing the alternative cassette exon selection between p(A)^+^ and p(A)^−^ RNA-seq data sets, circRNA-predominant alternative cassette exons were selected using stringent criteria. (*B*) The high-confidence circRNA-predominant cassette exons were determined with expression in at least two cell lines. (*C*) The strength of the 5′/3′ splice sites of high-confidence circRNA-predominant cassette exons (blue) was comparable with those of cassette exons identified in linear RNAs (light gray) and constitutive exons (dark gray). (ns) Not significant, (**) *P* value <0.01, Wilcoxon rank-sum test. (*D*) Similar densities of exonic splicing enhancers (ESE) were identified between high-confidence circRNA-predominant cassette exons (blue) and cassette exons identified in linear RNAs (light gray) and constitutive exons (dark gray). (ns) Not significant, (*) *P* value < 0.05, Wilcoxon rank-sum test. (*E*) The high-confidence circRNA-predominant cassette exons (blue) were slightly less conserved than were cassette exons identified in linear RNAs (light gray) and constitutive exons (dark gray).

In the current study, we focused on the analysis of alternative cassette exon inclusion/exclusion in circRNAs. A positive correlation was observed between the number of circRNAs and the number of circRNA-predominant cassette exons (Supplemental Fig. S8A). High-confidence circRNA-predominant cassette exons were further identified with a stringent pipeline (Fig 5A,B; Supplemental Fig. S8B). Genomic feature analysis revealed that the splice site strength and the density of different splicing regulators were not much different among these high-confidence circRNA-predominant cassette exons, constitutive exons, and the cassette exons that were identified in linear mRNAs (Fig. 5C,D; Supplemental Fig. S8C,D). However, circRNA-predominant cassette exons were generally less conserved than constitutive exons and cassette exons that were identified in linear mRNAs (Fig. 5E). It is unclear how these cassette exons could be predominantly spliced in circRNAs but not in their linear cognate RNAs.

### Novel circRNA-predominant cassette exons

**Figure 6.**
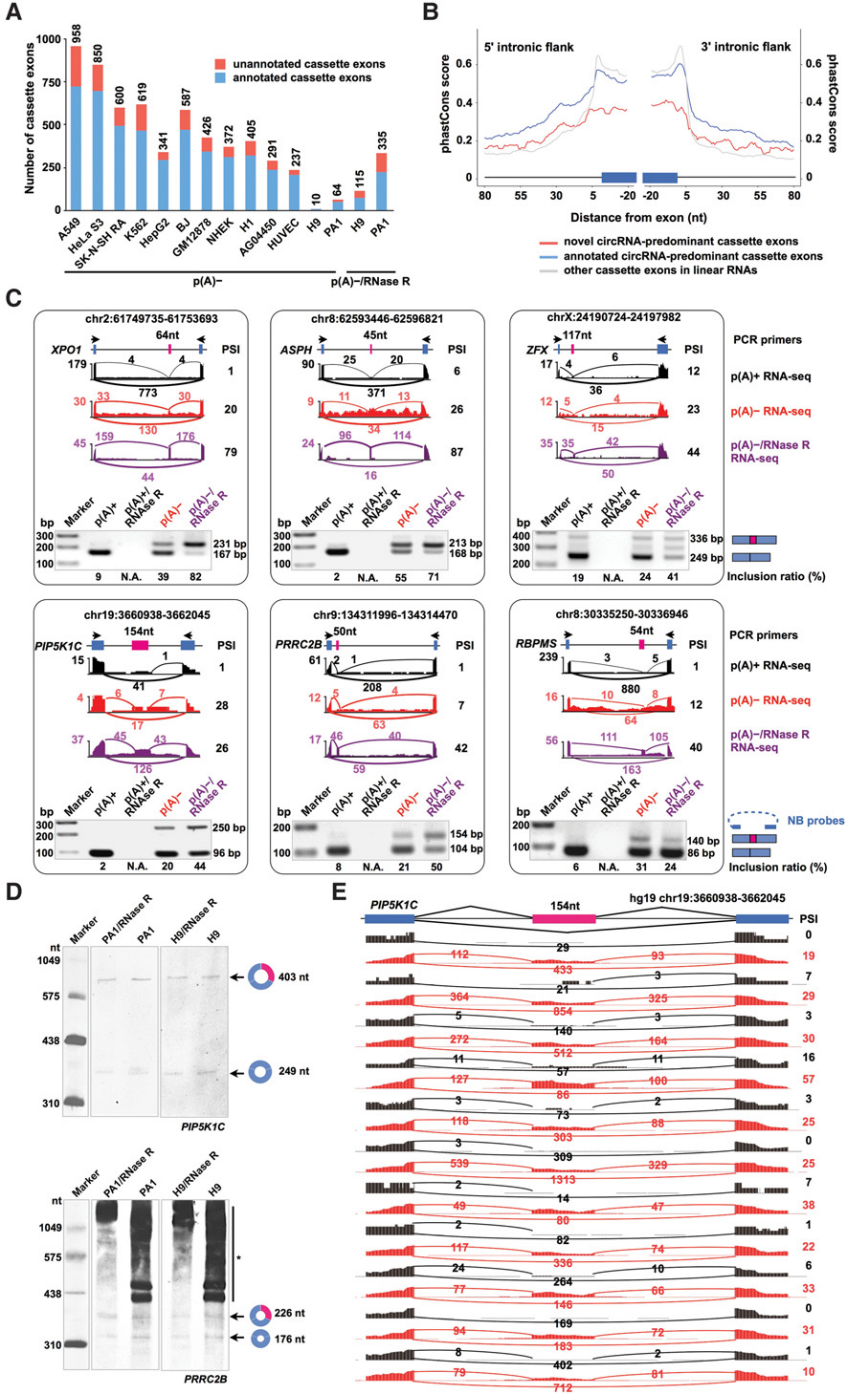
Unannotated circRNA-predominant alternative cassette exons. (*A*) Hundreds of previously unannotated circRNA-predominant cassette exons (red) were identified in circRNAs from individual cell lines. (*B*) Previously unannotated circRNA-predominant cassette exons (red) were much less conserved than the annotated circRNA-predominant cassette exons (blue) or cassette exons in linear RNAs (gray). (*C*) Validation of circRNA-predominant cassette exons in PA1 cells by RT-PCR. Similar to the RNA-seq results (PSI ratio), semiquantitative RT-PCR showed the detection of six circRNA-predominant cassette exons from the p(A)^−^ and p(A)^−^/RNase R RNA population but barely any from p(A)^+^ RNAs. The inclusion ratio of circRNA-predominant cassette exons from RT-PCR was determined by Quantity One (Bio-Rad). Note that circRNA-predominant cassette exons in *PIP5K1C*, *PRRC2B*, and *RBPMS* loci (*bottom*) were not previously annotated by RefGene, UCSC Known Genes, or Ensembl. Magenta bars, circRNA-predominant cassette exons. Blue bars, known exons. Divergent PCR primers were indicated as black arrows. (*D*) Validation of circRNA-predominant cassette exon inclusion from different cell lines by Northern blot on denaturing PAGE gels. The circRNAs with alternative cassette exon inclusion/exclusion in both *PIP5K1C* and *PRRC2B* loci were detected in PA1 and H9 cell lines. Note that the circRNA-predominant cassette exons in *PIP5K1C* or *PRRC2B* loci are previously unannotated. Magenta in the circles, circRNA-predominant cassette exons. Blue in the circles, known exons. (*) Linear RNA background. (*E*) Visualization of circRNA-predominant cassette exon inclusion in *PIP5K1C* locus from different cell lines. The inclusion ratio of the circRNA-predominant cassette exons from RNA-seq was indicated by PSI. Note that the circRNA-predominant cassette exon in the *PIP5K1C* locus is a newly identified exon in this study. Magenta bar, circRNA-predominant cassette exon. Blue bars, other exons.

Strikingly, we have identified hundreds to thousands of previously uncharacterized circRNA-predominant cassette exons (Fig. 6A; Supplemental Table S5), representing up to 25% of the highly expressed circRNAs in different cell lines. These circRNA-predominant novel cassette exons are much less conserved than are the annotated circRNA-predominant exons or other cassette exons that were present in linear RNAs (Fig. 6B). Examples of randomly selected circRNA-predominant cassette (novel) exons (Fig. 6C, bottom), are shown in both p(A)^−^ and p(A)^−^/RNase R RNA-seq data sets in PA1 cells (Fig. 6C) and validated by RT-PCR and/or by Northern blot in PA1 and H9 cells (Fig. 6C,D). Finally, circRNA-predominant cassette exons could be detected in multiple cell lines with different inclusion rates, as exemplified by the new circRNA-predominant cassette exon in the human *PIP5K1C* gene (Fig. 6E).

## Discussion

Different from canonical splicing that joins an upstream 5′ splice (donor) site with a downstream 3′ splice (acceptor) site, splicing also occurs in a reversed orientation (back-splicing), by which a downstream 5′ splice (donor) site links an upstream 3′ splice (acceptor) site, resulting in the production of circRNAs from back-spliced exons from pre-RNAs (Chen 2016). Interestingly, a single gene locus can produce multiple circRNAs through alternative 5′/3′ back-splice site selections that are uniquely identified in circRNAs (Fig. 1A; Zhang et al. 2014). In addition, it is well known that alternative splicing significantly contributes to expanding the complexity and diversity of transcriptomes. We concluded from the current study that this scenario is also the case for circRNA. Alternative splicing greatly expands the circRNA complexity (Fig. 1B), as the majority of human circRNAs consist of multiple exons.

With CIRCexplorer2, thousands of instances of alternative back-splicing and alternative splicing were found in circRNAs (Figs. 2, 5). Importantly, thousands of new circRNA-related exons and new cassette exons were identified (Figs. 4, 6). Interestingly, the alternative use of back-splice sites and canonical splice sites was strikingly diverse among different cell lines (Figs. 2, 5), which suggests that circRNA-related alternative back-splicing/splicing events are under regulation.

What factors could contribute to alternative back-splicing regulation? It has been reported that both *cis*-elements and *trans*-factors can facilitate back-splicing, presumably by bridging two back-splice sites close together to overcome the unfavorable catalysis by the spliceosome (Ashwal-Fluss et al. 2014; Liang and Wilusz 2014; Zhang et al. 2014; Chen and Yang 2015; Conn et al. 2015). Indeed, the majority of circRNAs that undergo alternative back-splicing contain paired complementary sequences in introns flanking both proximal and distal back-splice sites, and we further confirmed that the competition of RNA pairing results in alternative back-splicing by taking advantage of engineered expression vectors. Although the paired complementary sequences are presumably identical among all tested human cell lines, diverse alternative back-splicing landscapes were observed, suggesting that the regulation of alternative back-splicing is more complicated than the current depiction. Other factors, such as additional RNA binding proteins (RBPs) that are differentially expressed in various cell lines, may contribute to the selection of alternative back-splicing, resulting in the diverse regulation of alternative back-splicing among different cell lines. In fact, as RBP-mediated regulation of alternative splicing is prevalent (Nilsen and Graveley 2010), we suspect that back-splicing could be regulated by similar mechanisms.

It is also interesting to find all four basic types of alternative splicing patterns in circRNA production (Supplemental Fig. S7; Supplemental Methods). Strikingly, many alternatively spliced cassette exons appeared to be circRNA-predominant. Although the detailed mechanism is unclear, it is possible that such events could occur post-transcriptionally during circRNA biogenesis (Kramer et al. 2015; Zhang et al. 2016). Finally, the biological significance of circRNA-predominant alternative splicing awaits further investigation.

Since most circRNAs are expressed at low levels, RNase R, an enzyme that digests linear RNAs but preserves circRNAs (Suzuki et al. 2006), has been used to enrich circRNAs and to further verify the existence of circRNA-specific alternative splicing from both PA1 and H9 samples that were prepared in the lab (Fig. 6). However, p(A)^−^/RNaseR RNA-seq data sets were largely absent in publicly available ENCODE samples. Although we have applied the same stringent criteria (Supplemental Fig. S8B; Supplemental Methods) to annotate high-confidence circRNA-predominant alternative splicing, we cannot rule out higher rates of false-positives in these ENCODE samples due to the lack of RNase R treatment. Nevertheless, we assume that more circRNA-specific alternative (back-)splicing events could be further revealed with the addition of extra RNase R samples and the application of stringent methods used in this study.

Collectively, we concluded that alternative back-splicing and different types of alternative splicing in circRNAs are prevalent in human transcriptomes. The alternative back-splicing and circRNA-predominant alternative splicing events are highly diverse among different cell lines, indicating that additional *cis*-elements and *trans*-factors involved in circRNA biogenesis are yet to be identified. Finally, the involvement of thousands of previously uncharacterized exons during the alternative back-splicing/splicing of circRNAs suggests an even more complex landscape of RNA (back-)splicing and its regulation in human transcriptomes.

## Methods

### Upgraded CIRCexplorer2 pipeline

We have upgraded the previously reported computational pipeline CIRCexplorer (Zhang et al. 2014) to a new version (CIRCexplorer2) (Supplemental Methods; Supplemental Material) to comprehensively decipher the alternative back-splicing/splicing pattern of circRNAs in multiple cell lines (GEO: GSE26284, GSE24399, GSE60467, GSE48003, and GSE75733) (Djebali et al. 2012; Zhang et al. 2014).

### Characterization of alternative back-splicing

To systematically evaluate each alternative 5′/3′ back-splicing event, PCU was defined as in Figure 2B. High-confidence alternative 5′/3′ back-splicing events were further selected for more detailed characterization (Supplemental Table S2). Briefly, after clustering all circRNAs based on their 5′/3′ back-splice sites, circRNAs with highly expressed 5′/3′ back-splice sites (at least one circRNA with RPM ≥ 0.1) were selected for further analysis (Fig. 2A; Supplemental Fig. S4B).

### Characterization of alternative splicing in circRNAs

All four basic types of alternative splicing events were detected and quantitated in highly expressed circRNAs (RPM ≥ 0.1) from p(A)^−^ and/or p(A)^−^/RNase R RNA-seq data sets with relevant metrics (Supplemental Methods). At the same time, parallel p(A)^+^ RNA-seq data sets were aligned to the GRCh37/hg19 human reference genome by TopHat2 (Kim et al. 2013), and all relevant alternative splicing events in the linear RNAs were identified accordingly. By comparing alternative splicing between highly expressed circRNAs (RPM ≥ 0.1) and their linear cognates, all types of circRNA-specific/-predominant alternative splicing were determined with stringent criteria described in the Supplemental Methods.

### Cell culture, total RNA isolation, polyadenylated/nonpolyadenylated RNA separation, RNase R treatment, and RNA-seq

PA1 cells were cultured using standard protocol provided by ATCC. PA1 cells were grown in MEMα (Gibco) with 10% FBS and 1× GlutaMax (Gibco). H9 cells were maintained as described (Zhang et al. 2013). Total RNA isolation, polyadenylated/nonpolyadenylated RNA separation, RNase R treatment, and RNA-seq were performed as described (Yang et al. 2011; Zhang et al. 2013; Yin et al. 2015).

### RT-PCR, Northern blot, and Sanger sequencing

RT-PCR and Northern blots were used to evaluate the relative abundance of circRNAs as described (Zhang et al. 2013, 2014). PCR bands with novel circRNA-predominant exons were further subjected to Sanger sequencing. PCR primers and Northern blot probes are listed in Supplemental Table S6.

## Data access

Raw and processed RNA-seq data from this study have been submitted to the NCBI Gene Expression Omnibus (GEO; http://www.ncbi.nlm.nih.gov/geo/) under accession number GSE75733. Source code of CIRCexplorer2 is included in the Supplemental Material. Sanger trace files are available at NCBI Trace Archives (http://www.ncbi.nlm.nih.gov/Traces/home/index.cgi) with TI numbers from 2343264014 to 2343264040.

## Supplementary Material

Supplemental Material
