# Supplementary material for: Diverse alternative back-splicing and alternative splicing landscape of circular RNAs
Source: Genome Res. 2016 Sep;26(9):1277–87. doi: 10.1101/gr.202895.115 (PMC5052039; doi:10.1101/gr.202895.115)
Supplement: Supplemental Material [file supp_26_9_1277__index.html]

Diverse alternative back-splicing and alternative splicing landscape of circular RNAs — Supplemental Material 

# Diverse alternative back-splicing and alternative splicing landscape of circular RNAs

## Supplemental Material

- Supplemental\_Fig\_S1.pdf
- Supplemental\_Fig\_S2.pdf
- Supplemental\_Fig\_S3.pdf
- Supplemental\_Fig\_S4.pdf
- Supplemental\_Fig\_S5.pdf
- Supplemental\_Fig\_S6.pdf
- Supplemental\_Fig\_S7.pdf
- Supplemental\_Fig\_S8.pdf
- Supplemental\_Methods.pdf
- Supplemental\_Material.tar.gz
- Supplemental\_Table\_S1.pdf
- Supplemental\_Table\_S2.xlsx
- Supplemental\_Table\_S3.xlsx
- Supplemental\_Table\_S4.xlsx
- Supplemental\_Table\_S5.xlsx
- Supplemental\_Table\_S6.pdf
