# Supplementary material for: Diverse alternative back-splicing and alternative splicing landscape of circular RNAs
Source: Genome Res. 2016 Sep;26(9):1277–87. doi: 10.1101/gr.202895.115 (PMC5052039; doi:10.1101/gr.202895.115)
Supplement: Supplemental Material [file supp_gr.202895.115_Supplemental_Fig_S1.pdf]

A

Aligner	Algorithm	Compatible with Cufflinks	Memory consumption	# of circular RNAs with mapped fusion reads ≥ 1	# of circular RNAs with mapped fusion reads ≥ 2
STAR	suffix arrays	partial	~28G	circRNAs: 11,155	4,199
				ciRNAs: 1,521	334
segemehl	suffix arrays	poor	~70G	circRNAs: 12,871	5,030
				ciRNAs: 3,263	824
MapSplice	FM index	poor	~5G	circRNAs: 4,609	4,609
				ciRNAs: 86	86
TopHat 2 & TopHat-Fusion	FM index	perfect	~3G	circRNAs: 9,957	5,082
				ciRNAs: 983	327

B

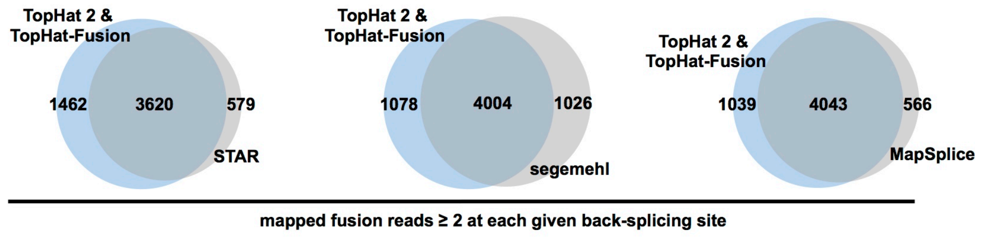

C

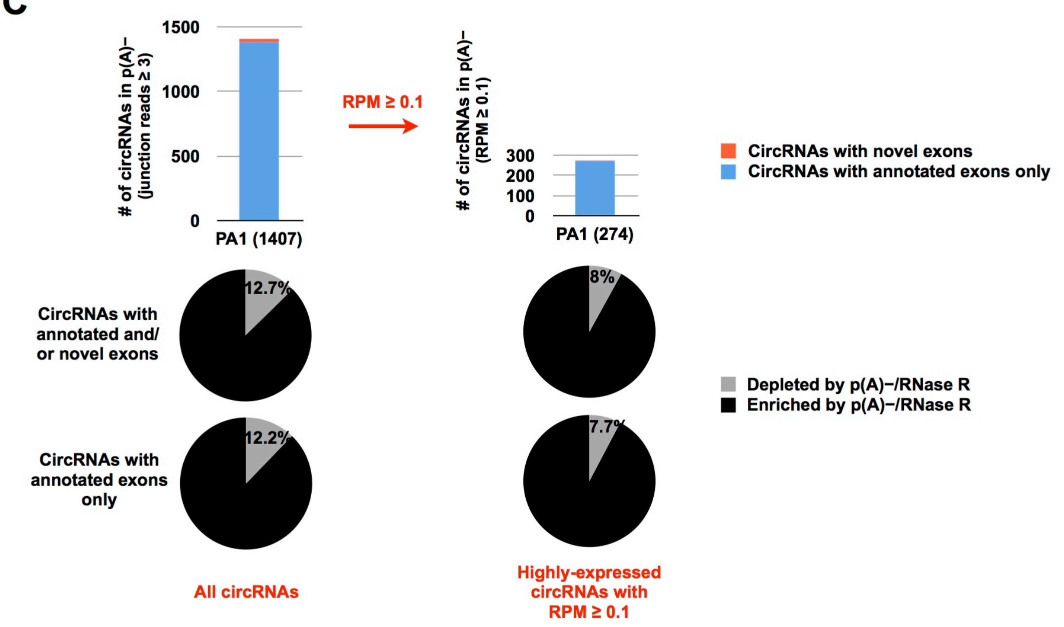

Supplemental Figure S1. Comparison of different aligners adopted in CIRCexplorer2.

(A) Summary of the different aligners that were applied for circRNA identification with the CIRCexplorer2 pipeline. Note that not only circRNAs but circular intronic RNA (ciRNAs) (Zhang et al. 2013) could be also predicted from all of the aligners.

(B) Comparison of detected circRNAs between different aligners. The best overlap was found between TopHat/TopHat-Fusion and MapSplice.

(C) The false discovery rate of upgraded CIRCexplorer2 pipeline remains at the low level. Note that over 85% of all or over 90% of highly-expressed circRNAs identified in p(A)− RNA-seq were enriched by RNase R treatment in p(A)−/RNase R RNA-seq in PA1 cells.
