# Supplementary material for: Diverse alternative back-splicing and alternative splicing landscape of circular RNAs
Source: Genome Res. 2016 Sep;26(9):1277–87. doi: 10.1101/gr.202895.115 (PMC5052039; doi:10.1101/gr.202895.115)
Supplement: Supplemental Material [file supp_gr.202895.115_Supplemental_Fig_S2.pdf]

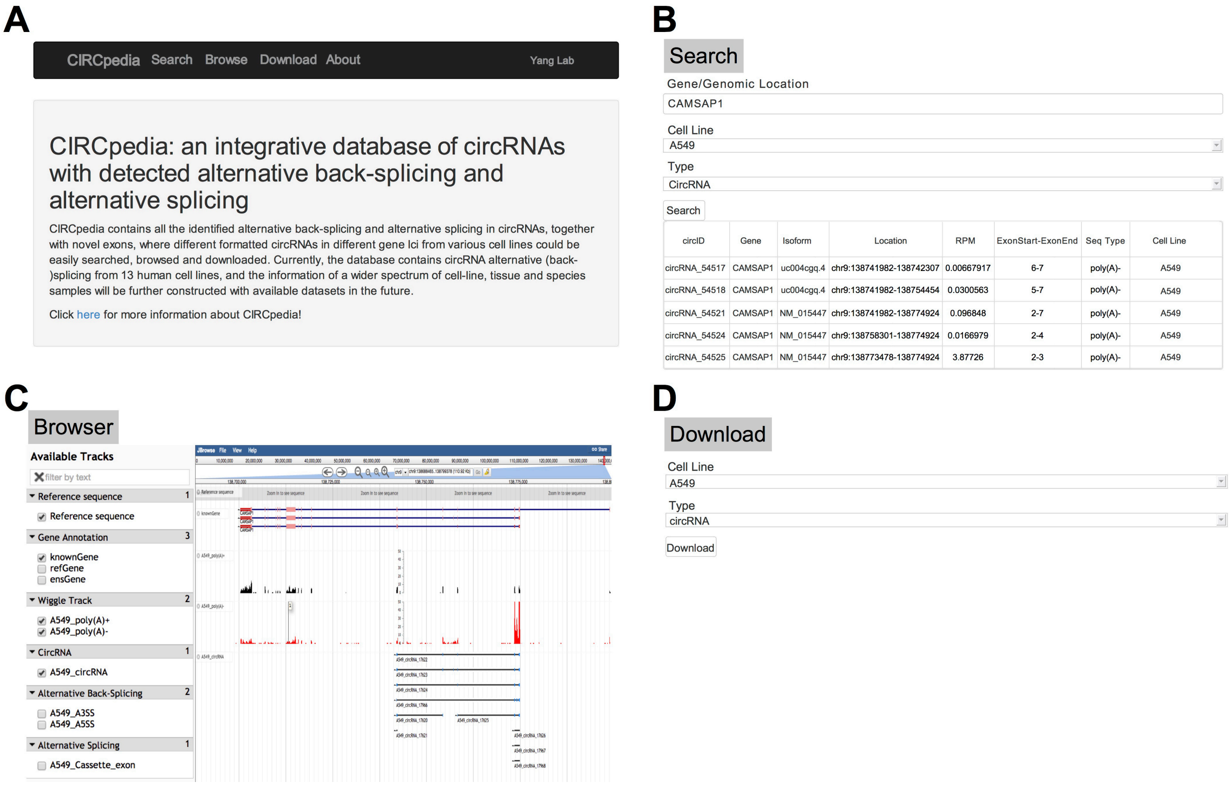

Supplemental Figure S2. CIRCpedia database

(A) The snapshot of the CIRCpedia database. All of the circRNAs produced from any individual gene locus in different cell lines can be easily searched, browsed and downloaded. Currently, the database contains circRNA back-splicing and alternative splicing from 13 human cell lines, and information on a wider spectrum of cell-line, tissue and species samples will be constructed when additional high-quality RNA-seq datasets are available.

(B) A simple search is available from the search page of CIRCpedia. CIRCpedia provides query support by gene symbols and genomic locations.

(C) Visualization of detected alternative back-splicing and alternative splicing in circRNAs in the website-embedded JBrowse.

(D) Tables for alternative back-splicing, alternative splicing and novel exons from each cell lines can be accessed from the download page.
