# Supplementary material for: Diverse alternative back-splicing and alternative splicing landscape of circular RNAs
Source: Genome Res. 2016 Sep;26(9):1277–87. doi: 10.1101/gr.202895.115 (PMC5052039; doi:10.1101/gr.202895.115)
Supplement: Supplemental Material [file supp_gr.202895.115_Supplemental_Fig_S3.pdf]

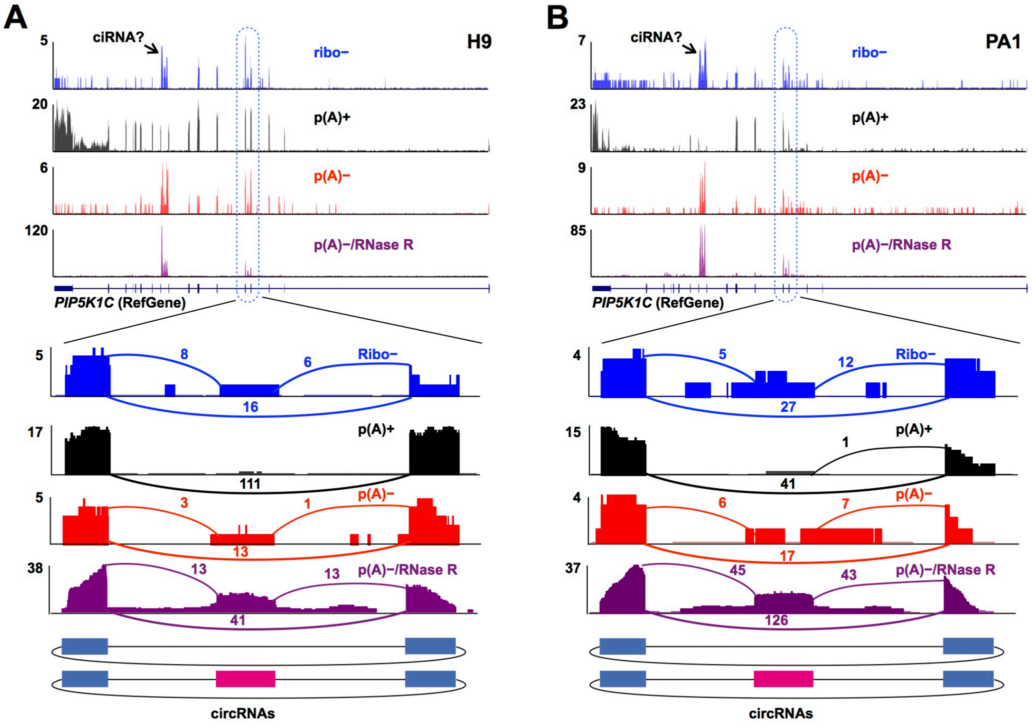

Supplemental Figure S3. The detection of circRNAs in the *PIP5K1C* locus from different RNA-seq datasets.

(A)(B) Identification and visualization of circRNAs in the *PIP5K1C* locus from H9 (A) and PA1 (B) cell lines. Different types of RNA-seq datasets from the ribo−, p(A)+, p(A)− or p(A)−/RNase R RNA population were used for comparison. Although circRNAs from the *PIP5K1C* locus could be determined from ribo−, p(A)− and p(A)−/RNase R RNA-seq datasets, ribo− RNA-seq is not suitable for studying the canonical splice events (cassette exon, Magenta bars) that occur specifically within circRNAs (See Fig. 1E for details). Blue bars, other exons. Black lines, introns. Red arc lines, back-splicing (circularization). Note that an additional ciRNA could be detected in the *PIP5K1C* locus.
