# Supplementary material for: Diverse alternative back-splicing and alternative splicing landscape of circular RNAs
Source: Genome Res. 2016 Sep;26(9):1277–87. doi: 10.1101/gr.202895.115 (PMC5052039; doi:10.1101/gr.202895.115)
Supplement: Supplemental Material [file supp_gr.202895.115_Supplemental_Fig_S4.pdf]

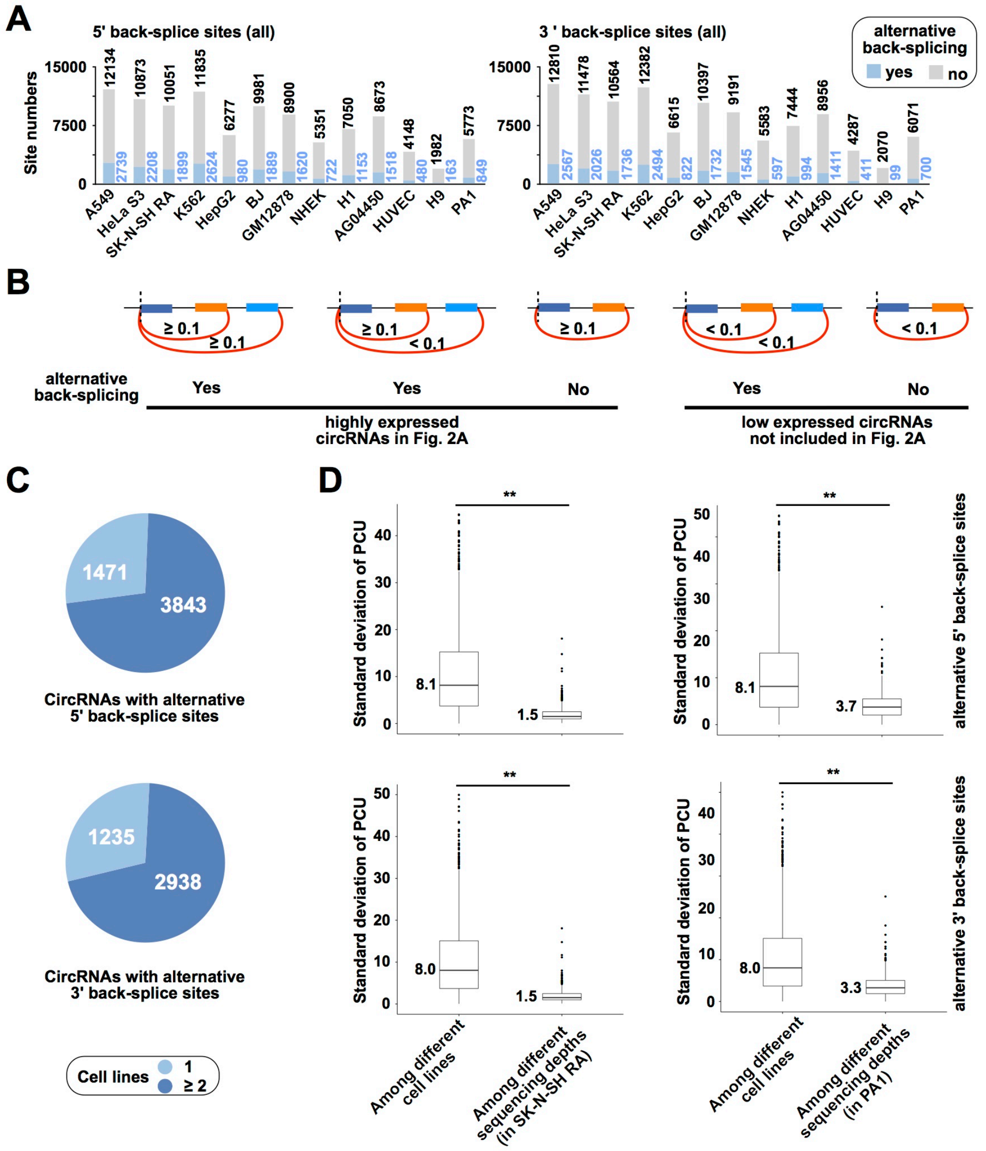

Supplemental Figure S4. Selection of high-confidence alternative back-splicing.

(A) Approximately 5-23% of back-splice sites were alternatively selected in circRNAs among the examined cell lines.

(B) A schematic diagram to show the selection of high-confidence alternative back-splicing with at least one of alternative 5'/3' back-splicing events was expressed with RPM ≥ 0.1.

(C) Approximately 70% of the alternative 5' (left panel) and 3' (right panel) back-splicing

events could be detected from multiple cell lines.

(D) The diverse usages of alternative back-splicing sites in highly-expressed circRNAs (with RPM ≥ 0.1 in at least three cell lines) were less affected by sequence depths than by their variable expression in different cell lines. Standard deviation of PCU was individually calculated among different cell lines or different sequencing depths in the same p(A)− RNA-seq samples. SK-N-SH RA p(A)− RNA-seq datasets (left) with different sequencing depths (100M, 150M, 200M, 250M, 300M, 350M and 400M) and PA1 p(A)− RNA-seq datasets (right) with different sequencing depths (20M, 30M, 40M, 50M, 60M, 70M and 80M) were carried out for comparison, respectively. ***p* value < 0.01, Wilcoxon rank-sum test.
