# Supplementary material for: Diverse alternative back-splicing and alternative splicing landscape of circular RNAs
Source: Genome Res. 2016 Sep;26(9):1277–87. doi: 10.1101/gr.202895.115 (PMC5052039; doi:10.1101/gr.202895.115)
Supplement: Supplemental Material [file supp_gr.202895.115_Supplemental_Fig_S5.pdf]

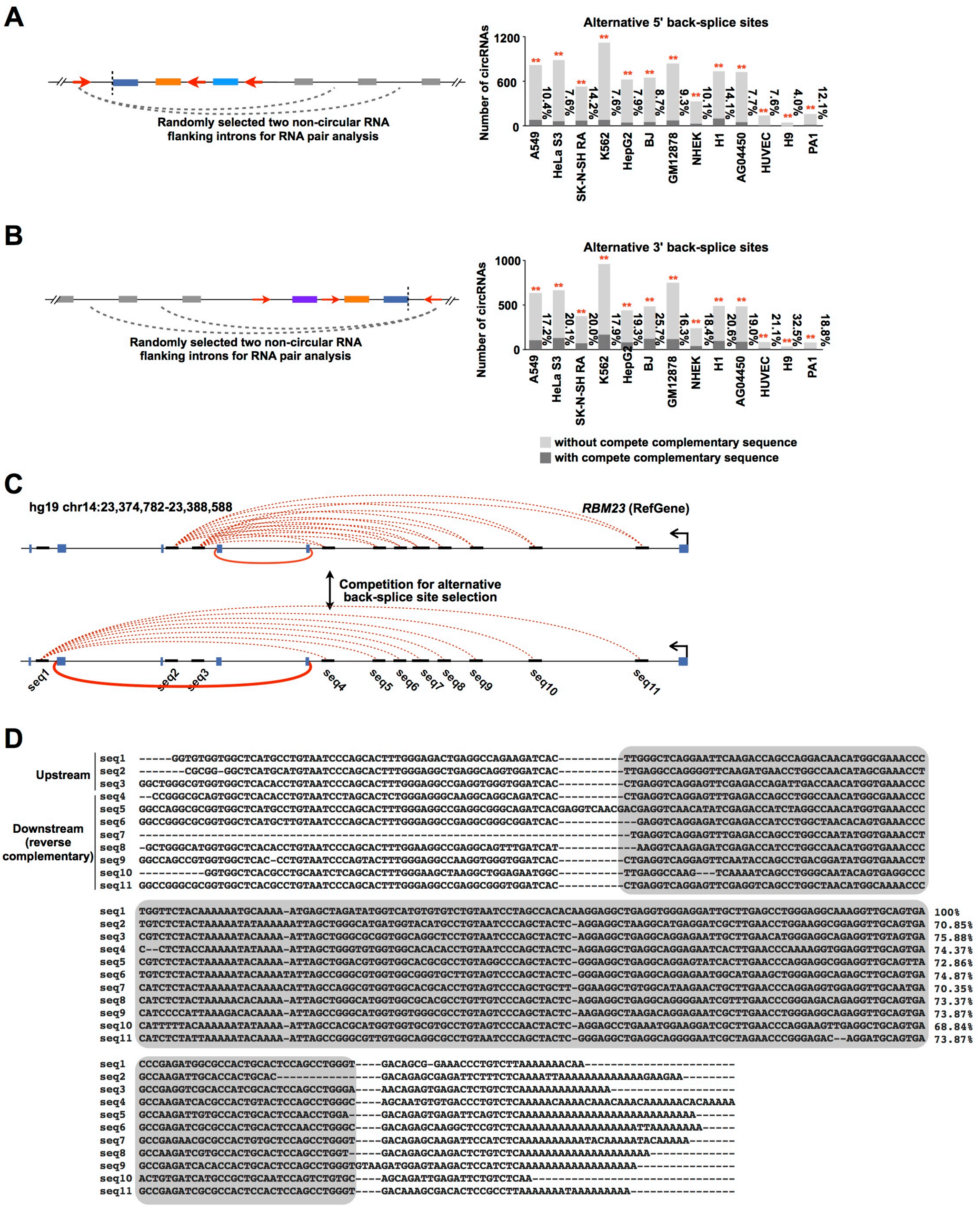

Supplemental Figure S5. Complementary sequence analysis in introns flanking either circularized or non-circularized exons.

(A)(B) Competition analysis of RNA pairs from randomly selected non-circularized-exon flanking introns in both alternative 5' (A) and 3' (B) back-splicing loci. Note that only about 20% of randomly selected intron pairs contain the potential paired complementary sequences for competition. **p value < 0.01, Fisher exact test, compared with corresponding sample in Figs. 3A and 3B.

(C) The competition of RNA pairs (red dashed arc lines) flanking proximal (top panel) or distal (bottom panel) back-splice sites possibly leads to alternative 5' back-splice site selection (red arc lines) in circRNAs from the human *RBM23* locus. Black bars, complementary sequences.

(D) Sequence analysis of upstream and downstream complementary sequences in human *RBM23* circRNA flanking introns. Note that the core regions (in grey shadow) of these upstream and downstream complementary sequences are similar to each other (up to over 70 % identity).
