# Supplementary material for: Diverse alternative back-splicing and alternative splicing landscape of circular RNAs
Source: Genome Res. 2016 Sep;26(9):1277–87. doi: 10.1101/gr.202895.115 (PMC5052039; doi:10.1101/gr.202895.115)
Supplement: Supplemental Material [file supp_gr.202895.115_Supplemental_Fig_S6.pdf]

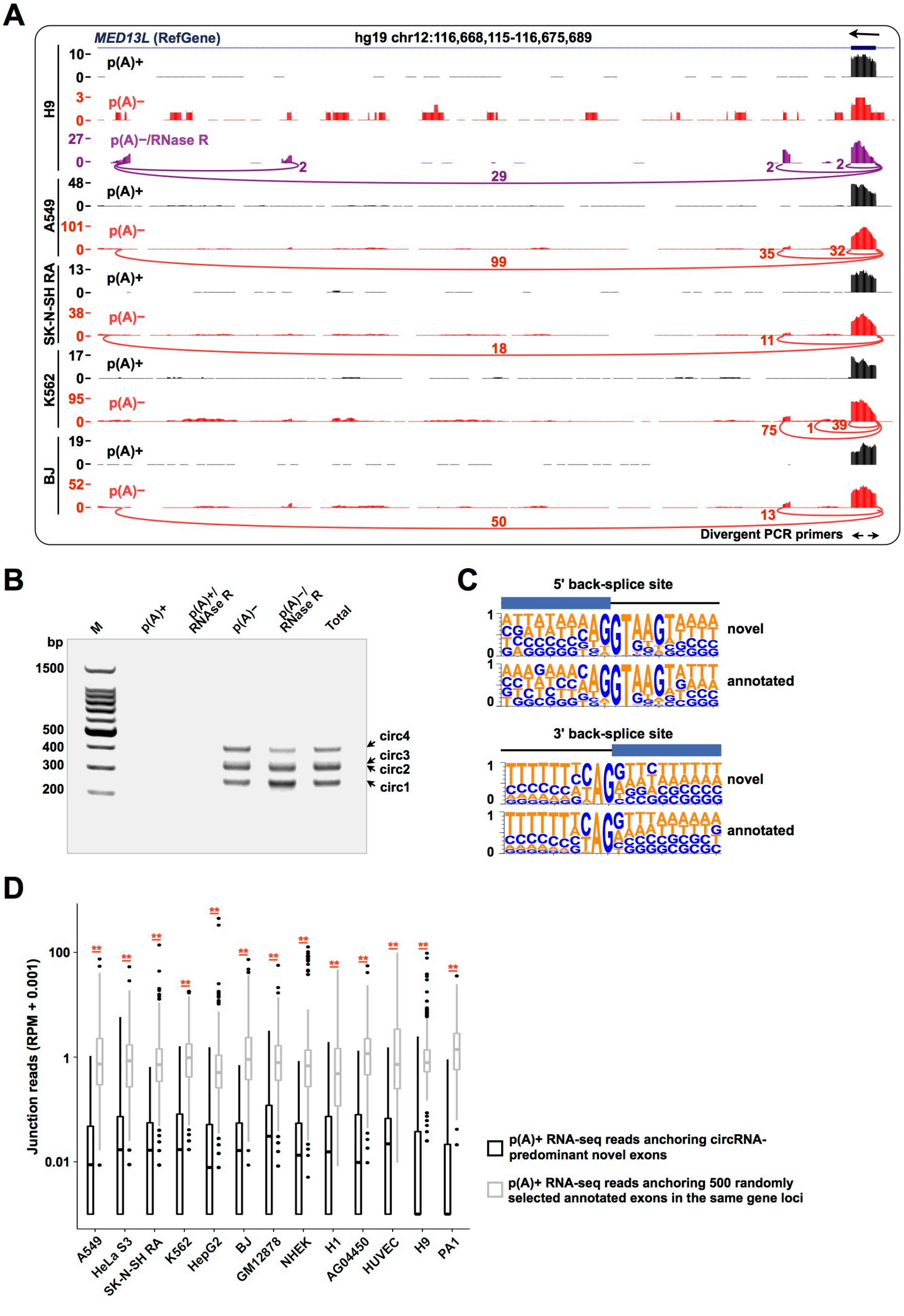

Supplemental Figure S6. Visualization and analysis of novel back-splicing sites.

(A) Multiple circRNAs with novel exons in the *MED13L* locus could be detected from multiple cell lines. Note that these novel exons were barely detected in the linear

counterparts from the paired p(A)+ RNA-seq (wiggle track in black). Black arrows, PCR primers.

(B) Novel exons from circRNAs were validated by RT-PCR with divergent primers (A) from p(A)− and p(A)−/RNase R RNA populations. Note that these novel exons were barely amplified in the linear counterparts from the p(A)+ RNA population. These novel exons in circRNAs were further confirmed by Sanger sequencing (Fig. 4A).

(C) Sequence feature analysis of both novel and annotated 5'/3' back-splice sites.

(D) CircRNA-predominant novel exons were less detected in linear RNAs. Note that fewer splicing junction reads could be detected in p(A)+ RNA-seq datasets to anchor these circRNA-predominant novel exons. While much more splicing junction reads could be found to anchor 500 randomly-selected annotated exons in the same gene loci. **p value < 0.01, Wilcoxon rank-sum test.
