# Supplementary material for: Diverse alternative back-splicing and alternative splicing landscape of circular RNAs
Source: Genome Res. 2016 Sep;26(9):1277–87. doi: 10.1101/gr.202895.115 (PMC5052039; doi:10.1101/gr.202895.115)
Supplement: Supplemental Material [file supp_gr.202895.115_Supplemental_Fig_S7.pdf]

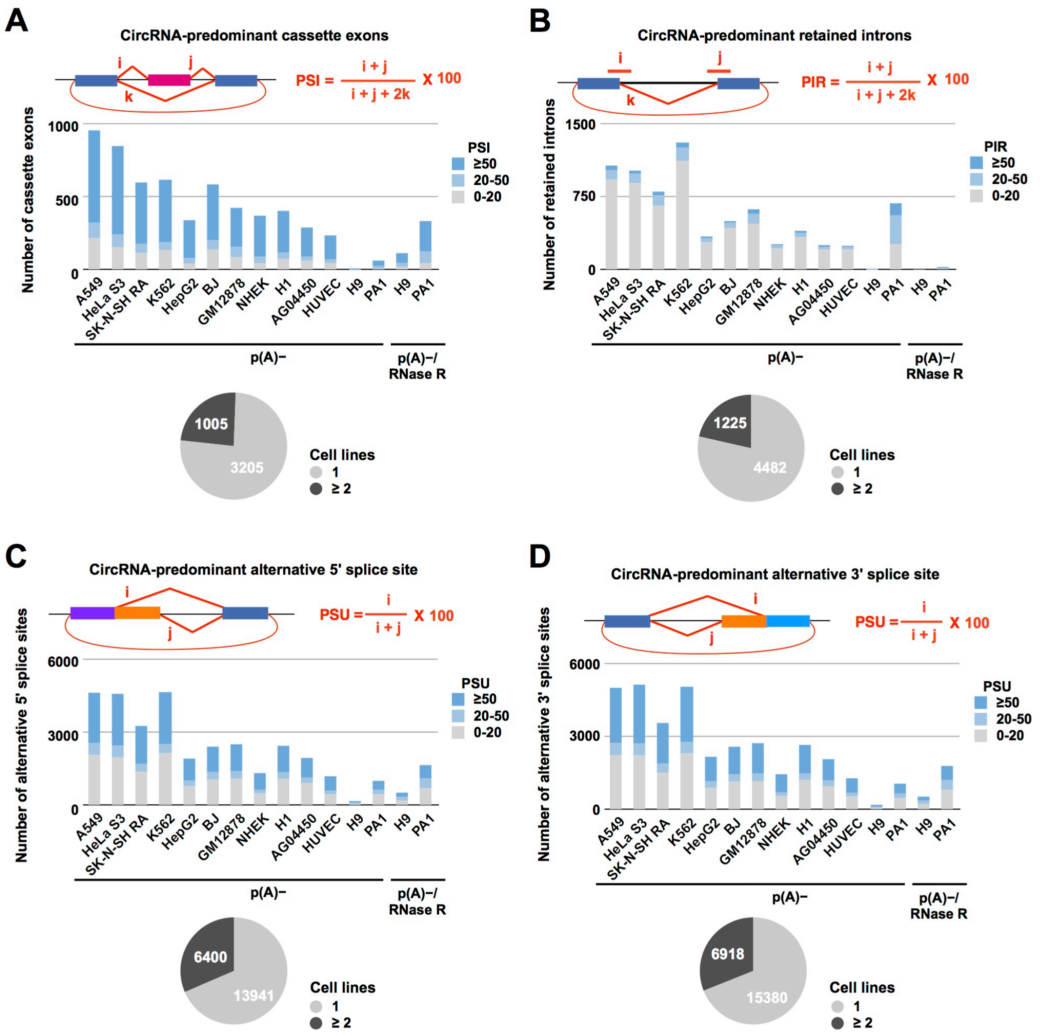

Supplemental Figure S7. The four types of alternative splicing in circRNAs.

(A)(B)(C)(D) All four basic types of alternative splicing could be identified in circRNAs from the examined cell lines. The extent of each alternative splicing event was quantitated by PSI (Percent Spliced In) for cassette exon inclusion/exclusion (A), PIR (Percent Intron Retention) for intron retention (B), and PSU (Percent Splice-site Usage) for alternative 5' splice site selection (C) and alternative 3' splice site selection (D). Note that 20-30% of alternative splicing events were detected in multiple examined cell lines (bottom panels).
