# Supplementary material for: Diverse alternative back-splicing and alternative splicing landscape of circular RNAs
Source: Genome Res. 2016 Sep;26(9):1277–87. doi: 10.1101/gr.202895.115 (PMC5052039; doi:10.1101/gr.202895.115)
Supplement: Supplemental Material [file supp_gr.202895.115_Supplemental_Fig_S8.pdf]

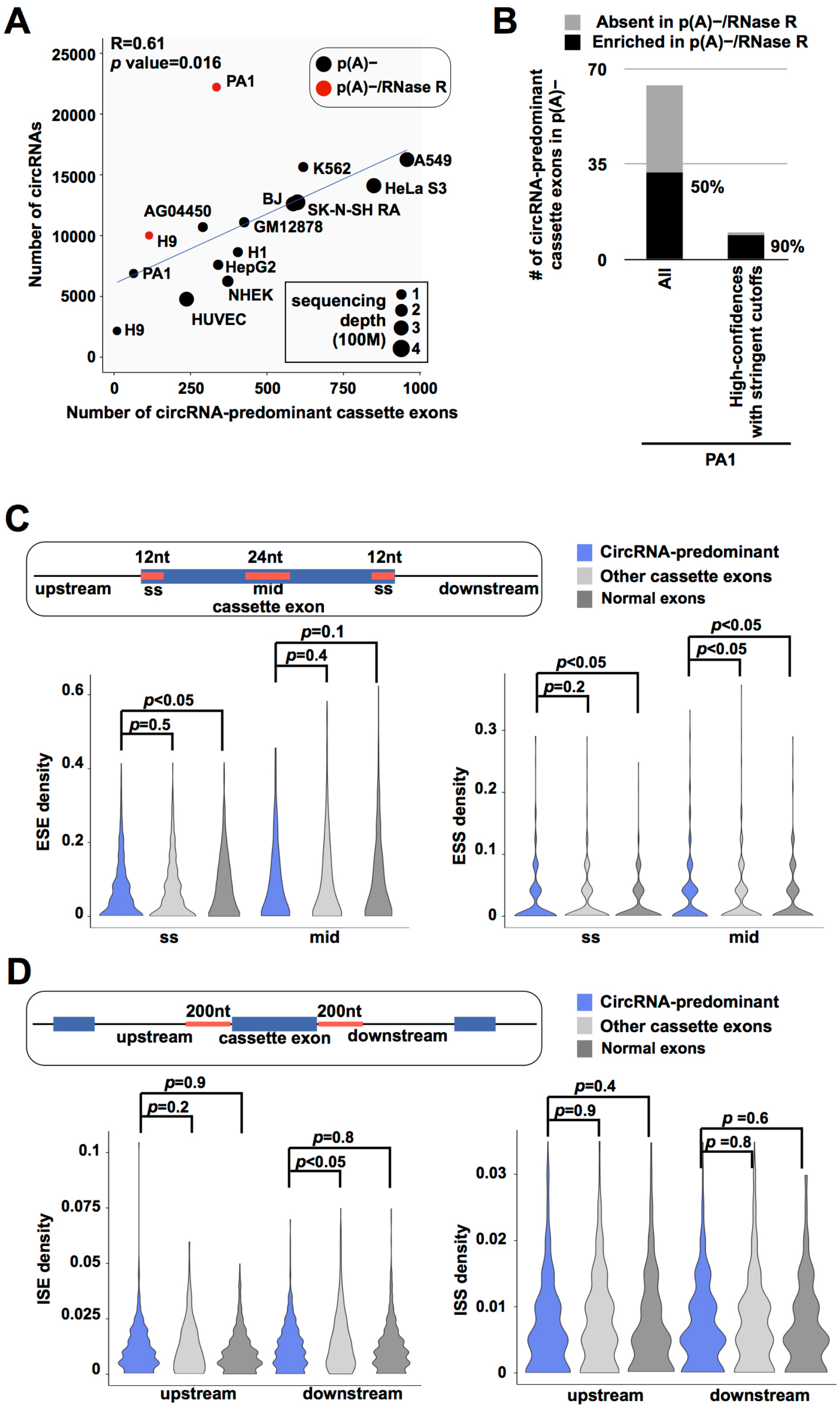

Supplemental Figure S8. Genomic feature analyses of circRNA-predominant cassette exons.

(A) The number of circRNA-predominant cassette exons was positively correlated with the number of detected circRNAs. The sequencing depth from different cell lines was indicated by different dot sizes.

(B) With the stringent cutoffs, about 90% of high-confidence circRNA-predominant cassette exons identified in p(A)− RNA-seq could be enriched by RNase R treatment in p(A)−/RNase R RNA-seq in PA1 cells.

(C) Exon splicing regulatory elements analysis. ESE (left panel) and ESS (right panel) of circRNA-predominant cassette exons, 500 randomly selected cassette exons only in linear RNAs and 500 randomly selected constitutive exons were analyzed and compared as previously reported (Methods). *p* value, Wilcoxon rank-sum test.

(D) Intronic splicing regulatory elements analysis. ISE (left panel) and ISS (right panel) of circRNA-predominant cassette exons, 500 randomly selected cassette exons only in linear RNAs and 500 randomly selected constitutive exons were analyzed and compared as previously reported (Methods). *p* value, Wilcoxon rank-sum test.
