# Supplementary material for: Diverse alternative back-splicing and alternative splicing landscape of circular RNAs
Source: Genome Res. 2016 Sep;26(9):1277–87. doi: 10.1101/gr.202895.115 (PMC5052039; doi:10.1101/gr.202895.115)
Supplement: Supplemental Material [file supp_gr.202895.115_Supplemental_Methods.pdf]

Supplemental Methods

Upgraded CIRCexplorer2 pipeline

To comprehensively decipher the alternative back-splicing/splicing pattern of circRNAs, we upgraded our previously reported computational pipeline CIRCexplorer (Zhang et al. 2014) to a new version (CIRCexplorer2). Several major improvements have been implemented in the upgraded pipeline. First, we have incorporated other aligners, such as STAR (Dobin et al. 2013), MapSplice (Jeck et al. 2013) and segemehl (Hoffmann et al. 2014), to fit the different requirements/preferences of RNA-seq mapping from different users. Second, poly(A)− RNA-seq reads that mapped to the genome and collinear exon-exon junctions were not simply discarded but were instead further *de novo* assembled to find novel exons and thus novel splicing events. Finally, TopHat-unmapped but TopHat-Fusion-mapped reads were realigned to both known and *de novo* assembled annotations to determine back-splice junctions from either annotated and/or novel exons (Fig. 1C). The application of *de novo* assembly leads to the discovery of a large number of novel exons. The source codes of CIRCexplorer2 can be accessed from https://github.com/YangLab/CIRCexplorer2, and the step-by-step usage is described as below.

Step 1: RNA-seq reads mapping with multiple aligners

CIRCexplorer2 makes full use of TopHat2/TopHat-Fusion for RNA-seq read mapping (Zhang et al. 2014). In addition, other aligners, such as STAR (Dobin et al. 2013), MapSplice (Jeck et al. 2013) and segemehl (Hoffmann et al. 2014), are also integrated into CIRCexplorer2 to meet different requirements for RNA-seq read mapping and data mining.

For the TopHat2/TopHat-Fusion pipeline, a two-step mapping strategy was exploited as previously described (Zhang et al. 2014), with the modifications suggested as below. Briefly, reads of human embryonic stem cell H9 with/without RNase R-treated poly(A)–/ribo– (poly(A)– for simplicity) RNA-seq (GEO:GSE48003, GEO:GSE24399 and GEO:GSE60467), human ovarian carcinoma PA1 with/without RNase R-treated poly(A)–/ribo– RNA-seq (GEO: GSE75733), or poly(A)–/ribo– RNA-seq of 11 ENCODE cell lines (GEO:GSE26284) (Supplemental Table S1) were first mapped using TopHat2 (Kim et al. 2013) (TopHat 2.0.9 with parameters: -g 1 --microexon-search -m 2) against the GRCh37/hg19 human reference genome with the UCSC Genes annotation (hg19 knownGene.txt updated at 2013/6/30). Unmapped reads were then extracted and aligned onto the GRCh37/hg19 human reference genome with TopHat-Fusion (Kim et al. 2013) (TopHat 2.0.9 with parameters: --fusion-search --keep-fasta-order --bowtie1 --no-coverage-search).

For other aligners, the same RNA-seq datasets were aligned using different aligners with respective parameters (STAR 2.4.0j with the following parameters: --chimSegmentMin 10; segemehl 0.2.0-418 with the following parameters: -S -M 1; and MapSplice 2.1.9 with the following parameters: -k 1 --non-canonical --fusion-non-canonical --min-fusion-distance 200).

Step 2: *de novo* assembly to annotate novel RNA transcripts/exons

The Cufflinks reference annotation based transcript (RABT) assembly method (Roberts et al. 2011) was used to identify new transcripts for circRNAs. In brief, TopHat2-mapped reads of poly(A)– RNA-seq were assembled using filtered gene annotations (gene annotations were collected from hg19 knownGene.txt updated at

2013/6/30, refFlat.txt updated at 2013/10/13 and ensGene.txt updated at 2014/4/6 and then filtered with at least two junction reads supporting all of the isoform junctions) using Cufflinks 2.2.1 with the following parameters: -u -F 0 -j 0. The assembled novel transcripts were combined with existing gene annotations (knownGene.txt, refFlat.txt and ensGene.txt) for later use.

Step 3: circRNA annotation with junction read re-alignments

Candidate back-splice junction reads (aligned on the same chromosome but in the non-collinear order) were extracted from the fusion alignment and re-aligned against combined (annotated and new) gene annotations to determine the precise positions of back-splice sites as previously described (Zhang et al. 2014). Note that all linear RNAs, including nascent linear RNAs, randomly degraded/deadenylated linear RNAs and spliced-out intermediates, were filtered out due to the lack of back-splicing junctions.

Similar strategy was employed as previously described (Hansen et al. 2016) to evaluate the false discovery rate of CIRCexplorer2. In brief, circRNAs identified in p(A)− RNA-seq datasets with at least three back-splicing junction reads were checked in corresponding p(A)−/RNase R RNA-seq datasets. For one specific circRNA, if the RPM of back-splicing junction reads in p(A)−/RNase R RNA-seq is higher than that in p(A)− RNA-seq, this circRNA is defined as enriched by RNase R. Otherwise, it is considered as depleted by RNase R. The false discovery rate of upgraded CIRCexplorer2 pipeline remains as low (Supplemental Fig. S1C) as that of CIRCexplorer (Hansen et al. 2016). It is worth noting that the false discovery rate analysis might also depend on many other factors, such as different sequencing depths and variable sequencing quality.

Characterization of alternative splicing in circRNAs

All four basic types of alternative splicing events, including alternative cassette exon selection, intron retention, alternative 5' splice site selection and alternative 3' splice site selection (Fig. 1B), were widely detected and quantitated in highly expressed circRNAs (RPM ≥ 0.1) from poly(A)– and/or poly(A)–/RNase R RNA-seq datasets with relevant metrics (alternative cassette exons: Percent Spliced In (PSI, Supplemental Fig. S7A) (Han et al. 2013; Irimia et al. 2014); intron retention: Percent Intron Retention (PIR, Supplemental Fig. S7B) (Braunschweig et al. 2014; Irimia et al. 2014); and alternative 5'/3' splice site selections: Percent Splice-site Usage (PSU, Supplemental Figs. S7C and S7D) (Irimia et al. 2014)). Because circRNAs lack poly(A) tails, we presumed that the splicing pattern in poly(A)– RNA-seq could represent the splicing landscape of circular RNAs and used the splicing pattern in poly(A)+ RNA-seq to evaluate the alternative splicing of linear RNAs. At the same time, parallel poly(A)+ RNA-seq datasets of relevant cell lines were aligned to the GRCh37/hg19 human reference genome by TopHat2 (Kim et al. 2013), and all of the relevant alternative splicing events in the linear RNAs were accordingly identified. By comparing alternative splicing between circRNAs and their linear cognates, all types of circRNA-specific/-predominant alternative splicing were determined based on the following criteria:

1. alternative cassette exons (Fig. 5A)

$P_{(\text{circular psi > linear psi, fisher exact test})} < 0.01$

Inclusion $\text{reads}_{\text{circular}} \geq 10$

Exclusion $\text{reads}_{\text{linear}} \geq 5$

High-confidence circRNA-predominant cassette exons were filtered as follows: 1) detected in at least two cell lines and 2) RPM ≥ 0.1 in at least one cell line in the current study. With these stringent cutoffs, about 90% of high-confidence circRNA-predominant cassette exons identified in PA1 p(A)− RNA-seq could be enriched by RNase R treatment in related p(A)−/RNase R RNA-seq (Supplemental Fig. S8B), suggesting a low false discovery rate (~10%) in our analyses. An optional filter (PSIcirc − PSIlinear ≥ 20% in at least one cell line) could be further applied to reduce the false discovery rate.

2. intron retention

Introns have no overlap with the annotated exons of any annotated genes.

Introns are covered by *de-novo*-assembled transcripts.

$PIR_{circular} > PIR_{linear}$

$P_{(exon\text{-}intron\ reads\ !=\ intron\ reads,\ binomial\ test)} \geq 0.05$

$E1I_{circular} + IE2_{circular} \geq 10$

$E1E2_{linear} \geq 5$

3. alternative 3' splice site selections

$PSU_{circular} > PSU_{linear}$

$0 < PSU_{circular} < 100\%$

Total splice site junction reads ≥ 5

4. alternative 5' splice site selections

$PSU_{circular} > PSU_{linear}$

$0 < PSU_{circular} < 100\%$

Total splice site junction reads ≥ 5

It is worth noting that intron retentions in circRNAs were frequently filtered out with RNase R treatment in PA1 and H9 cells (Supplemental Fig. S7B), which is very different to all the other three alternative splicing events that were enriched by RNase R (Supplemental Figs. S7A, S7C and S7D). It is possible that this type of circRNAs with retained intron were not stable with *in vitro* RNase R treatment, as previously reported (Zhang et al. 2014). In addition, the subgroup of circRNAs with retained introns have been recently reported to be mostly located in the nucleus (Li et al. 2015b), which is distinct from most other circRNAs that are located in the cytoplasm. It was suspected that the binding with protein cofactors in nucleus might stabilize these intron-exon circRNAs; however, the naked intron-exon circRNAs after purification is quite unstable with RNase R treatment.

CIRCpedia: an integrative database of circRNAs with detected alternative back-splicing and alternative splicing

All of the identified alternative back-splicing and alternative splicing events in circRNAs, together with newly identified exons, are available in the CIRCpedia database (http://www.picb.ac.cn/rnomics/circpedia). In this online database, multiple circRNAs produced from any individual gene locus in different cell lines can be easily searched, browsed and downloaded (Supplemental Fig. S2A). Currently, the database contains circRNA back-splicing and alternative splicing from 13 human cell lines, and information on a wider spectrum of cell-line, tissue and species samples will be constructed when additional high-quality RNA-seq datasets are available.

A simple search is available from the search page of CIRCpedia (Supplemental Fig. S2B). Users can easily query circRNA information in different cell lines and different types of alternative splicing or back-splicing. CIRCpedia provides query support by gene symbols and genomic locations. A specific gene symbol (/genomic location) will retrieve all of the circRNAs that have been identified in this gene locus (/genomic location), together with relevant alternative back-splicing and alternative splicing. In addition, users can also restrict their query to a specific type of alternative back-splicing/splicing or specific cell lines by different setting options. After the query, an informative table with genomic locations, circRNA ids, host gene names, relative expression, alternative (back)-splicing of relevant circRNAs and exon identity will be available to check online or download for further analysis. Useful links are also available to access more information or gene descriptions in GeneCard websites.

The detected alternative back-splicing and alternative splicing in circRNAs, together with the available gene annotation can be visualized in the website-embedded JBrowse (Skinner et al. 2009) (Supplemental Fig. S2C). Different types of tracks, such as gene annotation (including knownGene, refGene and ensGene), different RNA-seq tracks, alternative back-splicing/splicing of circRNAs, exon identity, and novel exons, are available to be visualized in the JBrowse.

Finally, tables for alternative back-splicing, alternative splicing and novel exons from each cell lines can be accessed from the download page (Supplemental Fig. S2D).

Splice site strength analysis

Splice site strength analysis was employed on novel back-splice sites using MaxEntScan (Yeo and Burge 2004). To better characterize novel back-splice sites, 500 annotated back-splice sites were randomly selected from annotated circRNAs as controls (Fig. 4D). A similar splice site strength analysis was performed with high-confidence circRNA-predominant cassette exons, 500 randomly selected cassette exons only in linear RNAs and 500 randomly selected constitutive exons (Fig. 5C).

GC content analysis

In sequences of approximately 150 nt upstream and downstream, 5'/3' novel and annotated back-splice sites were individually fetched and tiled up to compare the GC content (Fig. 4E).

Conservation analysis

Sequence conservation analysis was carried out by PhastCons metrics (Siepel et al. 2005). The PhastCons scores for multiple alignments of placental genomes were downloaded from UCSC, and the corresponding PhastCons scores of relevant regions were summarized to assess the conservation levels (Figs. 4F, 5E and 6B). The sequence conservation of novel/annotated back-splice sites (Fig. 4F), circRNA-predominant cassette exons (Fig. 5E) or novel/annotated circRNA-predominant cassette exons (Fig. 6B) was analyzed. A total of 500 randomly selected cassette exons only in linear RNAs and/or 500 randomly selected constitutive exons were used as controls (Figs. 5E and 6B).

Splicing regulatory element analysis

Sequences of previously established splicing regulatory elements (including ESE (Fairbrother et al. 2004), ESS (Wang et al. 2004), ISE (Wang et al. 2012) and ISS (Wang et al. 2013)) were examined from circRNA-predominant cassette exons, 500 randomly selected cassette exons only in linear RNAs and 500 randomly selected constitutive exons (Fig. 5D and Supplemental Figs. S8C and S8D). Briefly, splicing elements in the central 24 nt and 12 nt of the exonic sequences adjacent to the 5' and 3' splice sites (24 nt in total) (for ESE and ESS) or the flanking 200 nt intronic region around the 5' and 3' splice sites (for ISE and ISS) were individually analyzed and compared, as previously reported (Li et al. 2015a).

Complementary sequence analysis

For each highly expressed alternative 5'/3' back-splicing event (at least one circRNA with RPM ≥ 0.1, Fig. 2A), BLASTn (parameters: -word_size 11 -gapopen 5 -gapextend 2 -penalty -3 -reward 2) was used to detect complementary sequences (with a requirement of ≥ 50 nt) flanking the most proximal/distal back-splice sites. If both the most proximal circRNA and the most distal circRNA could be flanked by corresponding complementary sequences, this cluster of 5'/3' back-splicing events was defined as containing the feature of “competition of RNA pairs” (Figs. 3A and 3B).

To check whether the competition of RNA pairs flanking alternative 5'/3' back-splice sites is more frequent than expected by chance, control intron pairs were selected as described below. For each alternative 5' back-splicing event, two downstream non-circular RNA flanking introns were randomly selected to be individually

paired with the common upstream circRNA flanking intron. For each alternative 3' back-splicing event, two upstream non-circular RNA flanking introns were randomly selected to be individually paired with the common downstream circRNA flanking intron. This random selection of control intron RNA pairs was performed up to five times for each alternative 5'/3' back-splicing event. BLASTn (parameters: -word_size 11 -gapopen 5 -gapextend 2 -penalty -3 -reward 2) was then used to detect complementary sequences (with a requirement of ≥ 50 nt) within the control intron pairs.

Cell culture, plasmid construction, cell transfection, total RNA isolation, polyadenylated/non-polyadenylated RNA separation and RNase R treatment

Human ovarian carcinoma PA1 cells were cultured using standard protocol provided by ATCC. PA1 cells were grown in MEMα (Gibco) with 10% FBS and 1× GlutaMax (Gibco). Human embryonic stem cell line H9 cells were maintained as described previously (Zhang et al. 2013). Stem cell cultures were regularly evaluated for *POU5F1* expression every 3-4 weeks and cells were passaged every 6-7 days.

POLR2A circular RNA expression vectors with engineered complementary sequencing in two circRNA-flanking introns or one side of circRNA-flanking introns were obtained as described previously (Zhang et al. 2014). About 100 bp long complementary sequences were inserted into the middle intron between circRNA-residing exons by using BbvCI site with ClonExpress^{TM} II One Step Cloning Kit (Vazyme) (Fig. 3C). Primers for plasmid construction were listed in Supplemental Table S6. All the expression vectors were individually transfected into human HeLa-J cells

with X-tremeGENE 9 (Roche) according to the manufacturer's protocol. Total RNAs were extracted 24 hr after transfection.

Cultured cell lines with different treatments were harvested in Trizol (Pufei) and RNAs were extracted with Trizol Reagent (Pufei) according to the manufacturer's protocol, followed by DNase I treatment at 37 °C for 30 mins (DNA-freeTM kit, Ambion). Polyadenylated and non-polyadenylated RNA separation was carried out as described previously (Yang et al. 2011; Yin et al. 2015). RNase R treatment was carried out as described previously (Zhang et al. 2013). Briefly, purified RNAs were incubated with 40 U of RNase R (Epicentre) for 3 h at 37 °C and then were subjected to purification with Trizol.

RT-PCR, Sanger sequencing, Northern blot and RNA-seq

Each from 5 µg total RNAs, p(A)+, p(A)+/RNase R, p(A)–, or p(A)–/RNase R RNA sample was used for RT-PCR and/or Northern blot analyses as described previously (Zhang et al. 2013; Zhang et al. 2014). The first strand cDNA was transcribed with SuperScript III (Invitrogen) with random hexamers in a total volume of 20 µl, and 0.5 µl first-strand cDNA products were further amplified for 30 cycles (94 °C 30 sec, 55 °C 30 sec, 72 °C 20 sec) with 2×Taq Plus Master Mix (Vazyme) according to the manufacturer's protocol. PCR bands of individual novel circRNAs were further subjected to Sanger sequencing. Northern blots were carried out according to the manufacturer's protocol (DIG Northern Starter Kit, Roche). Briefly, RNAs were loaded on native Agarose or denaturing PAGE gels. Digoxigenin (Dig) labeled antisense and sense probes were made using T7 RNA polymerase by in vitro transcription with the

RiboMAX^{TM} Large Scale RNA Production Systems (Promega). PCR primers and Northern blot probes were listed in Supplemental Table S6.

RNA-seq libraries were prepared by using Illumina TruSeq Total RNA LT Sample Prep Kit (P/N 15026495) and subjected to deep sequencing with Illumina HiSeq 2000 at the CAS-MPG Partner Institute for Computational Biology Omics Core, Shanghai, China.

References

Braunschweig U, Barbosa-Morais NL, Pan Q, Nachman EN, Alipanahi B, Gonatopoulos-Pournatzis T, Frey B, Irimia M, Blencowe BJ. 2014. Widespread intron retention in mammals functionally tunes transcriptomes. *Genome Res* 24(11): 1774-1786.

Dobin A, Davis CA, Schlesinger F, Drenkow J, Zaleski C, Jha S, Batut P, Chaisson M, Gingeras TR. 2013. STAR: ultrafast universal RNA-seq aligner. *Bioinformatics* 29(1): 15-21.

Fairbrother WG, Yeo GW, Yeh R, Goldstein P, Mawson M, Sharp PA, Burge CB. 2004. RESCUE-ESE identifies candidate exonic splicing enhancers in vertebrate exons. *Nucleic Acids Res* **32**: W187-190.

Han H, Irimia M, Ross PJ, Sung HK, Alipanahi B, David L, Golipour A, Gabut M, Michael IP, Nachman EN et al. 2013. MBNL proteins repress ES-cell-specific alternative splicing and reprogramming. *Nature* 498(7453): 241-245.

Hoffmann S, Otto C, Doose G, Tanzer A, Langenberger D, Christ S, Kunz M, Holdt LM, Teupser D, Hackermuller J et al. 2014. A multi-split mapping algorithm for circular RNA, splicing, trans-splicing and fusion detection. *Genome Biol* 15(2): R34.

Irimia M, Weatheritt RJ, Ellis JD, Parikshak NN, Gonatopoulos-Pournatzis T, Babor M, Quesnel-Vallieres M, Tapial J, Raj B, O'Hanlon D et al. 2014. A highly conserved program of neuronal microexons is misregulated in autistic brains. *Cell* 159(7): 1511-1523.

Jeck WR, Sorrentino JA, Wang K, Slevin MK, Burd CE, Liu J, Marzluff WF, Sharpless NE. 2013. Circular RNAs are abundant, conserved, and associated with ALU repeats. *RNA* 19(2): 141-157.

Kim D, Pertea G, Trapnell C, Pimentel H, Kelley R, Salzberg SL. 2013. TopHat2: accurate alignment of transcriptomes in the presence of insertions, deletions and gene fusions. *Genome Biol* 14(4): R36.

Li YI, Sanchez-Pulido L, Haerty W, Ponting CP. 2015a. RBFOX and PTBP1 proteins regulate the alternative splicing of micro-exons in human brain transcripts. *Genome Res* **25**(1): 1-13.

Li Z, Huang C, Bao C, Chen L, Lin M, Wang X, Zhong G, Yu B, Hu W, Dai L et al. 2015b. Exon-intron circular RNAs regulate transcription in the nucleus. *Nat Struct Mol Biol* 22(3): 256-264.

Roberts A, Pimentel H, Trapnell C, Pachter L. 2011. Identification of novel transcripts in annotated genomes using RNA-Seq. *Bioinformatics* 27(17): 2325-2329.

Skinner ME, Uzilov AV, Stein LD, Mungall CJ, Holmes IH. 2009. JBrowse: a next-generation genome browser. *Genome Res* **19**(9): 1630-1638.

Wang Y, Ma M, Xiao X, Wang Z. 2012. Intronic splicing enhancers, cognate splicing factors and context-dependent regulation rules. *Nat Struct Mol Biol* **19**(10): 1044-1052.

Wang Y, Xiao X, Zhang J, Choudhury R, Robertson A, Li K, Ma M, Burge CB, Wang Z. 2013. A complex network of factors with overlapping affinities represses splicing through intronic elements. *Nat Struct Mol Biol* **20**(1): 36-45.

Wang Z, Rolish ME, Yeo G, Tung V, Mawson M, Burge CB. 2004. Systematic identification and analysis of exonic splicing silencers. *Cell* **119**(6): 831-845.

Yang L, Duff MO, Graveley BR, Carmichael GG, Chen LL. 2011. Genomewide characterization of non-polyadenylated RNAs. *Genome Biol* 12(2): R16.

Yeo G, Burge CB. 2004. Maximum entropy modeling of short sequence motifs with applications to RNA splicing signals. *J Comput Biol* **11**(2-3): 377-394.

Yin QF, Chen LL, Yang L. 2015. Fractionation of non-polyadenylated and ribosomal-free RNAs from mammalian cells. *Methods Mol Biol* 1206: 69-80.

Zhang XO, Wang HB, Zhang Y, Lu X, Chen LL, Yang L. 2014. Complementary sequence-mediated exon circularization. *Cell* 159(1): 134-147.

Zhang Y, Zhang XO, Chen T, Xiang JF, Yin QF, Xing YH, Zhu S, Yang L, Chen LL. 2013. Circular intronic long noncoding RNAs. *Mol Cell* 51(6): 792-806.
