# Supplementary material for: Diverse alternative back-splicing and alternative splicing landscape of circular RNAs
Source: Genome Res. 2016 Sep;26(9):1277–87. doi: 10.1101/gr.202895.115 (PMC5052039; doi:10.1101/gr.202895.115)
Supplement: Supplemental Material [file supp_gr.202895.115_Supplemental_Table_S1.pdf]

Cell line	Sequence type	GEO number	Total reads	TopHat mapping Readr	Percentage (%)	TopHat-Fusion mapping reads	Percentage (%)	Total mapping reads	Percentage (%)
A549	p(A)−	GSE26284	344,982,676	296,698,822	86.00	2,739,553	0.79	299,438,375	86.80
HeLa S3	p(A)−	GSE26284	357,743,456	275,252,894	76.94	3,140,035	0.88	278,392,929	77.82
SK-N-SH RA	p(A)−	GSE26284	413,917,186	343,762,576	83.05	3,328,957	0.80	347,091,533	83.86
K562	p(A)−	GSE26284	128,755,210	105,936,592	82.28	892,258	0.69	106,828,850	82.97
HepG2	p(A)−	GSE26284	136,674,012	105,537,480	77.22	932,780	0.68	106,470,260	77.90
BJ	p(A)−	GSE26284	339,926,812	277,528,646	81.64	2,907,444	0.86	280,436,090	82.50
GM12878	p(A)−	GSE26284	138,915,054	103,806,988	74.73	910,180	0.66	104,717,168	75.38
NHEK	p(A)−	GSE26284	184,805,618	118,090,812	63.90	967,351	0.52	119,058,163	64.42
H1	p(A)−	GSE26284	125,838,298	98,029,952	77.90	614,166	0.49	98,644,118	78.39
AG04450	p(A)−	GSE26284	122,314,760	103,131,773	84.32	932,414	0.76	104,064,187	85.08
HUVEC	p(A)−	GSE26284	374,319,422	303,859,279	81.18	2,102,096	0.56	305,961,375	81.74
H9	p(A)−	GSE24399<br>GSE60467	59,998,105	46,784,520	77.98	1,216,143	2.03	48,000,663	80.00
PA1	p(A)−	GSE75733	87,675,979	80,607,304	91.94	1,453,082	1.66	82,060,386	93.60
H9	p(A)−/ RNaseR	GSE48003	41,342,095	24,258,271	58.68	525,836	1.27	24,784,107	59.95
PA1	p(A)−/ RNaseR	GSE75733	69,931,871	64,506,214	92.24	756,820	1.08	65,263,034	93.32

Supplemental Table S1. RNA-seq datasets used in this study, including information of cell line, deep sequencing type, GEO accession number and alignment for each dataset.
