# Supplementary material for: Diverse alternative back-splicing and alternative splicing landscape of circular RNAs
Source: Genome Res. 2016 Sep;26(9):1277–87. doi: 10.1101/gr.202895.115 (PMC5052039; doi:10.1101/gr.202895.115)
Supplement: Supplemental Material [file supp_gr.202895.115_Supplemental_Table_S6.pdf]

Supplemental Table S6

(A) Primer sequences used to detect novel back-spliced exons in *MED13L* locus.

Primer name	Sequences
MED13L-novel-circRNA-F	GTGTATGGCGTCGTGATGTC
MED13L-novel-circRNA-R	CCCATGCCCTCCAAAATTGT

(B) Primer sequences used to validate circRNA-predominant cassette exons.

Primer name	Sequences
XPO1-F	TGGCTCAAGAAGTACTGACACA
XPO1-R	TTCGCACTGGTTCCTTGGAA
ASPH-F	CAAGAATGGGAGGAAAGGCG
ASPH-R	TCTCCATCACCATCAGCATCA
ZFX-F	ACTCATTTTTTGATGCAACAG
ZFX-R	GCTCAGGAATGATGACCGTT
PIP5K1C-F	ACTTCTACGTGGTGGAGAGC
PIP5K1C-R	GTGCATAGGTCTTGAAGCGG
PRRC2B-F	TTGCAGAAACCGACACAGTC
PRRC2B-R	CCTTCGTGTCCTACTGGCTT
RBPMS-F	TGAGGGTTCTCTTATAAAGCTCACA
RBPMS-R	TCTTTGCAGCCTCTGCTTCT

(C) Probes for Northern blots.

Probe name	Sequences
MED13L probe F	GCTGAACTCACGGGAATCAA
MED13L probe F (T7)	GAAATTAATACGACTCACTATAGGGA GGTTCATCTCCCCACCAGAA
PIP5K1C probe F	CGAAGGCAGCAACCTCACCCCCG
PIP5K1C probe R (T7)	GAAATTAATACGACTCACTATAGGGA CTGGGGAAGAAGATGCTCTCCAC
PRRC2B probe F	AATACAAATTCAGTGCCAGGTG
PRRC2B probe R (T7)	GAAATTAATACGACTCACTATAGGGA CTCCTGACTGATTGACTGTGTC
POLR2A probe F	CAGACTTTGACGGGGATGAGATG
POLR2A probe R (T7)	GAAATTAATACGACTCACTATAGGGA TGGGAGAATGCGGACCCGATGC

(D) Primer sequences used to *POLR2A* circRNA expression vectors.

Primer name	Sequences
Complementary 100 bp F	TTAGGTCAGTCCCTGGCTGACTTTTCAGT TTACAAAGGAC
Complementary 100 bp R	ATTCCAGCCTGCTTCCCTCATTCAACCTC TTTCTGCTCAA
